# Pod indehiscence in common bean is associated with the fine regulation of *PvMYB26*

**DOI:** 10.1093/jxb/eraa553

**Published:** 2020-11-28

**Authors:** Valerio Di Vittori, Elena Bitocchi, Monica Rodriguez, Saleh Alseekh, Elisa Bellucci, Laura Nanni, Tania Gioia, Stefania Marzario, Giuseppina Logozzo, Marzia Rossato, Concetta De Quattro, Maria L Murgia, Juan José Ferreira, Ana Campa, Chunming Xu, Fabio Fiorani, Arun Sampathkumar, Anja Fröhlich, Giovanna Attene, Massimo Delledonne, Björn Usadel, Alisdair R Fernie, Domenico Rau, Roberto Papa

**Affiliations:** 1 Dipartimento di Scienze Agrarie, Alimentari e Ambientali, Università Politecnica delle Marche, via Brecce Bianche, Ancona, Italy; 2 Max Planck Institute of Molecular Plant Physiology, Am Müehlenberg, Potsdam-Golm, Germany; 3 Dipartimento di Agraria, Università degli Studi di Sassari, Via E. De Nicola, Sassari, Italy; 4 Centro per la Conservazione e Valorizzazione della Biodiversità Vegetale, Università degli Studi di Sassari, SS 127bis, km 28.500 Surigheddu, Alghero, Italy; 5 Center of Plant Systems Biology and Biotechnology, Plovdiv, Bulgaria; 6 Scuola di Scienze Agrarie, Forestali, Alimentari ed Ambientali, Università degli Studi della Basilicata, viale dell’Ateneo Lucano, Potenza, Italy; 7 Dipartimento di Biotecnologie, Università degli Studi di Verona, Cà Vignal, Strada Le Grazie, Verona, Italy; 8 Plant Genetics Group, Agri-Food Research and Development Regional Service (SERIDA), Asturias, Spain; 9 Key Laboratory of Molecular Epigenetics of the Ministry of Education (MOE), Northeast Normal University, Changchun, China; 10 Institute of Biosciences and Geosciences (IBG-2): Plant Sciences, Forschungszentrum Jülich GmbH, Jülich, Germany; 11 University of Trento, Italy

**Keywords:** Common bean, convergent evolution, gene expression, genome-wide association study, introgression lines, *MYB26*, *Phaseolus vulgaris* L, pod anatomy, pod shattering

## Abstract

In legumes, pod shattering occurs when mature pods dehisce along the sutures, and detachment of the valves promotes seed dispersal. In *Phaseolus vulgaris* (L)., the major locus *qPD5.1-Pv* for pod indehiscence was identified recently. We developed a BC_4_/F_4_ introgression line population and narrowed the major locus down to a 22.5 kb region. Here, gene expression and a parallel histological analysis of dehiscent and indehiscent pods identified an *AtMYB26* orthologue as the best candidate for loss of pod shattering, on a genomic region ~11 kb downstream of the highest associated peak. Based on mapping and expression data, we propose early and fine up-regulation of *PvMYB26* in dehiscent pods. Detailed histological analysis establishes that pod indehiscence is associated with the lack of a functional abscission layer in the ventral sheath, and that the key anatomical modifications associated with pod shattering in common bean occur early during pod development. We finally propose that loss of pod shattering in legumes resulted from histological convergent evolution and that it is the result of selection at orthologous loci.

## Teaser

Linkage mapping and histological and expression pattern analyses in pods identified *PvMYB26* as the best candidate gene for pod indehiscence, mediated by a non-functional abscission layer in the pod.

## Introduction

Loss of seed shattering is a paradigmatic example of the changes that have occurred to crop plant traits compared with their wild progenitors, which collectively constitute the ‘domestication syndrome’ (Hammer, 1984). In wild species, specialized seed dispersal strategies are of fundamental importance for plant survival and fitness. Conversely, in domesticated forms, loss or reduction of seed shattering is desired to reduce yield losses.

Due to its complex evolutionary history, common bean (*Phaseolus vulgaris* L.) is an excellent model to study the domestication process (Bitocchi *et al.*, 2017), which included its parallel domestication in the Andes and Mesoamerica (Bitocchi *et al.*, 2013). In *P. vulgaris*, the dry beans are characterized by different degrees of pod shattering. These represent the majority of the domesticated pool (Gepts and Debouck, 1991), where a limited level of pod shattering has been conserved to favour the threshing of the dry pods. Variations in the pod shattering intensity are also associated with the environmental conditions during maturation (e.g. humidity and temperature) (Parker *et al.*, 2020).

Secondary domestication events have resulted in the development of totally indehiscent snap bean cultivars, with a dominance of the Andean gene pool among commercial snap beans (Wallace *et al.*, 2018). Snap beans are suitable for green pod production due to the low fibre content in the pod walls and sutures (i.e. the stringless type). Pioneering investigations into *Arabidopsis thaliana* (L.) have reconstructed the genetic pathways associated with its fruit differentiation and silique shattering, which provides a model of the mechanisms underlying seed dispersal for other crop species (for a review, see Di Vittori *et al.*, 2019). In common bean, Koinange *et al.* (1996) identified the qualitative locus *St* on chromosome Pv02 for the presence of the pod suture string in a recombinant inbred line (RIL) population derived from the cross between an Andean snap bean (i.e. Midas) and a wild Mesoamerican genotype (i.e. G12873). Their observation that pod fibre content correlates with pod shattering was confirmed by Murgia *et al.* (2017), who identified an association between the carbon and lignin contents and modulation of pod shattering. Nanni *et al.* (2011) and Gioia *et al.* (2013) identified the orthologous genes of *AtSHP* (Liljegren *et al.*, 2000) and *AtIND* (Liljegren *et al.*, 2004), respectively, in common bean, where *PvIND* was co-mapped with *St* (Koinange *et al.*, 1996). However, *PvSHP* and *PvIND* did not show any polymorphic sequences associated with occurrence of pod shattering (Nanni *et al.*, 2011; Gioia *et al.*, 2013). Recently, Rau *et al.* (2019) identified a major locus on chromosome Pv05 for pod indehiscence (*qPD5.1-Pv*) on an introgression line (IL) population that was obtained by the initial backcross between the Andean snap bean landrace Midas (totally indehiscent) and the highly shattering RIL MG38 previously developed by Koinange *et al.* (1996). The same locus was confirmed by Parker *et al.* (2020) who performed a genome-wide association study (GWAS) on an Andean diversity panel. Rau *et al.* (2019) thus proposed a model in which at least three additional hypostatic loci on chromosomes Pv04, Pv05, and Pv09 are involved in modulation of pod shattering, with multifactorial inheritance of the trait previously suggested by Lamprecht (1932). The recent identification of a major locus for pod shattering in common bean (Rau *et al.*, 2019) and in cowpea (Lo *et al.*, 2018) in a syntenic region on chromosome Pv05 supports the occurrence of convergent molecular evolution in legume species. Moreover, Parker *et al.* (2020) suggested that the gene orthologous to *GmPDH1* in soybean (Funatsuki *et al.*, 2014) is involved in the resistance to pod dehiscence in accessions from the race Durango, compared with the more susceptible accessions from race Mesoamerica within the Mesoamerican pool.

In the present study, we developed a population of 1197 BC_4_/F_4_ ILs by backcrossing six highly shattering ILs from Rau *et al.* (2019) with the recurrent parent Midas. The population was dedicated to pod-shattering syndrome traits, with the aim to narrow down the major locus *qPD5.1-Pv*, and to promote recombination at quantitative trait loci (QTLs) for pod shattering. We also performed differential expression analysis at the transcriptome level (i.e. RNA-seq) between wild and domesticated pods, and at the major locus *qPD5.1-Pv* for target genes [i.e. Real-time quantitative reverse transcription PCR (Real-time qRT–PCR)], using a comparison of indehiscent and highly dehiscent pods from near isogenic lines (NILs). The expression analysis for the putative structural genes of lignin biosynthesis and a parallel histological analysis of the indehiscent and dehiscent pods allow reconstruction of the main phenotypic events associated with the modulation of pod shattering that occur early during pod development. Finally, we propose several candidate genes with potential roles in the modulation of pod shattering, both at the genome-wide level and at known QTLs.

## Materials and methods

### Development of the introgression line population

Here, we developed an IL population (1197 BC_4_/F_4_) for the mapping of pod-shattering traits (Supplementary Fig. S1). The IL population was developed starting from a cross between the domesticated Andean variety Midas, as ‘stringless’ and totally indehiscent, and the highly shattering wild Mesoamerican genotype G12873, to provide an initial set of RILs (Koinange *et al.*, 1996). One RIL (i.e. MG38) showed high shattering, wild traits of the seeds and pods, a determinate growth habit, and the absence of photoperiod sensitivity, so it was selected as a donor parent for pod-shattering traits for backcrossing with the recurrent Midas (BC_1_). Overall, three backcrosses were performed using Midas as the recurrent parent and performing phenotypic selection for high shattering for each backcrossed generation, which provided 70 ILs from BC_3_/F_4_:F_5_ families, and 217 ILs from BC_3_/F_6_:F_7_ families (Murgia *et al.*, 2017; Rau *et al.*, 2019). In the present study, six highly shattering ILs were selected as the donor parents for high pod shattering, and were further backcrossed (BC_4_) with Midas, providing six subpopulations (BC_4_/F_1_ families), for the lines 232B (from a BC_3_/F_4_:F_5_ family) and 244A/1A, 038B/2A2, 038B/2C1, 038A/2D1, and 038B/2B1 (from BC_3_/F_6_:F_7_ families). Seeds of BC_4_/F_1_ individuals and of the seven parental lines were pre-germinated in Petri dishes using deionized water. The plants were individually grown in the greenhouse of the Dipartimento di Scienze Agrarie, Alimentari ed Ambientali at the Polytechnic University of Marche in Ancona, Italy, between January and May 2016. BC_4_/F_2_ seeds were collected from 100 BC_4_/F_1_ lines, and 1353 BC_4_/F_2_ harvested seeds were planted in an open field at Villa D’Agri, Marsicovetere, Potenza, Italy in the summer of 2016. Some of these (636 BC_4_/F_2_ seeds) were pre-germinated using vermiculite and deionized water, and the seedlings were transplanted on the first planting (7 June 2016), while the other 717 BC_4_/F_2_ seeds were directly sown as a second planting (26 July 2016). The pods were collected from 942 BC_4_/F_2_ ILs in October 2016. The BC_4_/F_3_ plants were obtained by single seed descent and grown in the greenhouse between February and May 2017. With the aim to reach an initial population size of 1000 BC_4_/F_3_ individuals, two BC_4_/F_3_ seeds were sown from a few dehiscent BC_4_/F_2_ lines. The pods were collected from 724 BC_4_/F_3_ individuals. Then 2230 BC_4_/F_4_ seeds and 109 seeds from the seven parental lines of the new population were sown in an open field at Villa D’Agri in the summer of 2017. The seeds were directly sown on 22 June, and additional sowing was performed to recover any missing plants. One BC_4_/F_4_ seed from each BC_4_/F_3_ indehiscent line, and at least four BC_4_/F_4_ seeds from each BC_4_/F_3_ dehiscent line were sown, with the aim to promote segregation and recombination at the major locus *qPD5.1-Pv* for pod indehiscence on Pv05 (Rau *et al.*, 2019), at which a recessive domesticated allele determines the totally indehiscent phenotype only in the homozygous condition. The pods were collected from 1197 BC_4_/F_4_ ILs. The BC_4_/F_2_ experimental field scheme provided 12 rows, with a sowing distance of 0.6 m and 1.5 m within and between the rows, respectively. The BC_4_/F_4_ field scheme consisted of 2339 holes across nine rows, with a sowing distance of 0.25 m and 1.2 m within and between the rows, respectively. In the field trials, the ILs were completely randomized within the six BC_4_/F_1_ families. Weed control was provided using a mulching plastic sheet, and pest control treatments were with Ridomil Gold (fungicide) and Klartan 20 Ew (against aphids). The plants were watered daily using an automatic irrigation system, and fertilization with nitrogen, phosphorus, and potassium was applied before tillage.

### Phenotyping of the introgression line population

Phenotyping for pod shattering was performed in the field trials both qualitatively (i.e. occurrence of pod shattering, with each plant classified as dehiscent if it showed at least one shattered pod), and quantitatively, by assigning a score to each dehiscent line based on the pod twisting: 0 (no twisted pods per plant); 1 (1% <twisted pods <10%); 2 (≥10% <twisted pods <24%); and 3 (≥24% of twisted pods). Shattering was evaluated in the BC_4_/F_2_ ILs across four dates (Supplementary Table S1) until the uniform ripening of the entire plants, and in the BC_4_/F_4_ lines across two main dates (18 October and 22 October), plus two additional dates (26 October and 12 November) for plants which were not fully ripened at the earlier dates. Pod shattering was also evaluated post-harvest by examination of the completely dry pods. For the BC_4_/F_1_ individuals, each genotype was classified as easy to thresh (i.e. pods opened very easily along sutures), similar to the highly shattering parents, or as totally indehiscent, similar to the domesticated parent Midas. For the other experiments, phenotyping was performed by testing the resistance to opening when the ripened pods were subjected to increasing manual pressure directly on the sutures, according to the scoring system in Supplementary Table S2. Moreover, a comprehensive phenotypic trait for pod shattering was assigned manually to each BC_4_/F_4_ line (i.e. ‘SH y/n’; presence or absence of pod dehiscence), which combined field and post-harvest phenotyping.

### Genotyping and genome-wide association study for pod shattering

Young leaves were collected from 1197 BC_4_/F_4_ ILs and 55 replicates from the seven parental lines that were grown during the last IL field experiment. The leaves were dried within 12 h of collection using silica gel. Genomic DNA (gDNA) was extracted from the leaves using the Exgene Plant SV kit (Geneall Biotechnology) and stored at –20 °C. The gDNA integrity was determined on 1% agarose gels, and the DNA quality was measured using a photometer (NanoPhotometer NP80; Implen) and quantified with the dsDNA assay kit (Qubit HS; Life Technologies). The gDNA concentrations were adjusted to 25 ng µl^–1^, and the genotyping was performed using genotype-by-sequencing (GBS) (Elshire *et al.*, 2011) by Personal Genomics (Verona, Italy). The protocol for the GBS library preparation is provided below, according to the procedure reported in Rau *et al.* 2019. For each sample, 200 ng of gDNA were digested for 2 h at 75 °C with 1.25 U of *Ape*KI (New England Biolabs, NEB) in 1× NEB 3.1 buffer, in a final volume of 20 μl. The results of the digestion were verified by running the digested DNA and the intact gDNA on a 4200 TapeStation using the Genomic DNA assay (Agilent Technologies). The digested DNA was ligated to a double-stranded barcoded-adaptor (previously annealed, 0.05 µM final concentration) with 1 U of T4 DNA ligase (Invitrogen) in the presence of 1× ligase buffer in a final volume of 50 µl. A total of 24 different barcoded-adaptors were employed to uniquely identify 24 samples at a time (Supplementary Table S3). The ligation reaction was performed in a thermocycler for 10 min at 30 °C, and 4 h at 22 °C (unheated lid), followed by inactivation for 30 min at 65 °C (heated lid). The samples were subsequently pooled (25 μl from each sample; 24 samples with different barcoded-adaptors) and purified using beads (0.4× AMPure XP; Beckman Coulter) following the manufacturer’s instructions. The purified pool was resuspended in 30 µl of water. The DNA fragments with the desired length were selected using a BluePippin system (Sage Science). The 30 µl of the purified pool were loaded in a 1.5% Agarose Dye-Free cassette (internal standard, 250 bp–1.5 kb DNA size range) and run with a tight mode set to 550 bp. The eluted size-selected pool (~40–50 µl) was brought to a volume of 60 µl with water. Half of the purified and size-selected pool (30 µl) was subsequently amplified in a 50 µl reaction volume using 1 U of Taq Phusion polymerase in the presence of 1× Taq Phusion HF buffer, 0.3 mM dNTPs, and three different primers: Primer MP1 (0.5 µM), Primer MP2 (0.01 µM), and PPIX Illumina Index (0.5 µM), the latter including an index for Illumina sequencing. A total of eight PPI Illumina Index primers with eight different Illumina indexes were utilized, allowing a multiplexing of eight pools (=192 samples) at a time. Primer sequences are reported in Supplementary Table S4. 

Amplification was performed following the PCR programme of: 30 s at 98 °C, 18 cycles of 10 s at 98 °C, 30 s at 65 °C, and 30 s at 72 °C, and 5 min at 72 °C for final elongation. Final GBS libraries were purified with beads (1.5× AMPure XP; Beckman Coulter). The size distribution of final GBS libraries was determined on a 4200 TapeStation using a D1000 Assay (the average size distribution expected was 560 bp). Supplementary Fig. S2 shows the final GBS library structure. The final GBS libraries were quantified by qPCR using primer annealing on the Illumina adaptor sequences (Supplementary Table S4), and on the basis of a reference standard curve. The GBS libraries were sequenced [HiSeqX platform; Illumina with 2× 150 bp reads mode at Macrogen Inc. (South Korea)], which generated 1.5 million fragments per sample on average. The sequencing reads were demultiplexed based on their barcodes. Adaptors and low-quality bases in the FASTQ files were removed using the Cutadapt software, version 1.8.3 (Martin, 2011). The filtered reads were aligned to the reference genome of *P. vulgaris* 442 version 2.0 using the BWA-mem software, version 0.7.17-r1188 (Li and Durbin, 2009). The resulting BAM files were realigned using the GATK RealignerTargetCreator and IndelRealigner software, version 3.8.1, to remove errors. Variant calling was performed for all of the samples together, using the GATK UnifiedGenotyper software, version 3.8.1 (McKenna *et al.*, 2010), and the variants were filtered based on GATK best practice. 

The raw single nucleotide polymorphism (SNP) dataset (2 419 927 SNPs) was checked for quality, and loci with missing data >95% and with minor allele frequency (MAF) <0.05 were excluded from further analysis. Additionally, filtering was performed to remove SNPs that were either missing in one parental set (i.e. MIDAS or MG38), monomorphic between parents, or located in SCAFFOLDS (as SCAFFOLDS were not associated with any of the investigated traits). The dataset was then imputed for missing data by using beagle.5 (Browning *et al.*, 2018). A further filtering was performed after imputation to remove a few more sites that were monomorphic between the parents. The final dataset included 1253 individuals (i.e. 1196 BC_4_/F_4_ ILs, 55 parental lines of the BC_4_ population, and the references Midas and MG38) and 19 420 SNPs. GWAS was performed by using the mixed linear model (MLM) as implemented in the rMVP package (https://github.com/xiaolei-lab/rMVP). Overall, seven descriptors of pod shattering were considered for GWAS analysis from the three main phenotypic datasets [i.e. Sh y/n (integration of field and post-harvest data), field, and post-harvest]: Sh y/n (dehiscent versus indehiscent lines), Sh y/n after filtering (18 lines that showed signs of diseases and/or a low pod production were removed), Sh y/n including lines with an intermediate phenotype between the dehiscent and the indehiscent, Field (presence versus absence of pod shattering), Field (percentage of twisting pods per plant), Post-harvest (putative dehiscent versus putative indehiscent), Post-harvest (quantititative; mapping of all the phenotypic classes 0, 1, 1.5, 2, 3).

### RNA sequencing and differential gene expression analysis

The wild dehiscent Mesoamerican genotype G12873 and the fully indehiscent Andean variety Midas were grown for the collection of their pods under controlled conditions in a growth chamber at the Institute of Biosciences and Geosciences (IBG-2, Forschungszentrum Jülich) in 2014. Two plants were planted for both the G12873 and Midas genotypes. In the same experiment, a total of 57 plants were grown from 43 different genotypes, as for 14 of these two replicates were available. As regards the overall number of plants, nine were Andean domesticated, 18 were Andean wild (AW), 12 were Mesoamerican domesticated (MD), and 18 were Mesoamerican wild (MW). Moreover, three of the nine Andean domesticated were snap bean types (AD_Snap; totally indehiscent), while the other six were dry beans (AD), according to the phenotypic data and the available information. The list of the accessions is provided in Supplementary Data S1. The experimental conditions were 24/20 °C day/night temperature, 70% relative air humidity, photon flux density of 400–500 μmol m^–2^ s^–1^, and short-day photoperiod conditions (10/14 h light/dark). Fertilization was provided for N-K-P and trace elements. 

The pods were collected for each genotype at 5 days after pod setting (DAP) and 10 DAP. These were snap-frozen in liquid nitrogen before storage at –80 °C. After RNA extraction, the cDNA libraries were prepared according to the Illumina TruSeq RNA LT protocol, and the RNA sequencing was performed with the HiSeq paired-end V4/4000 125/150 cycles sequencing technology. Sequencing was performed by the Genomics and Microarray Core Laboratory at the University of Colorado in Denver (USA), and the raw data quality check and alignment were performed by Sequentia Biotech (Barcelona, Spain). The read quality checking was performed on the raw sequencing data using the FastQC tool, and low-quality portions of the reads were removed using BBduk. The minimum length of the reads after trimming was set to 35 bp, and the minimum base quality score to 25. High quality reads were aligned against the *P. vulgaris* reference genome (G19833 genome v2.1; http://phytozome.jgi.doe.gov/) using the STAR aligner software, version 2.5.0c. The reads that could not be aligned against the first reference genome were mapped against the second reference genome (*P. vulgaris* L., BAT93; Vlasova *et al.*, 2016). FeatureCounts, version 1.4.6-p5, was used to calculate the gene expression values as raw read counts. Normalized TMM values (trimmed means of M-values) were also calculated. Here, the raw reads data were used to perform the differential gene expression analysis across the two developmental stages, using the DESeq2 package (Love *et al.*, 2014) in R (R Core Team, 2019). The differential gene expression was calculated for each gene (as log_2_ fold change), and the *P*-values were adjusted according to the Benjamini–Hochberg procedure (Benjamini and Hochberg, 1995). Differential gene expression was performed at 5 DAP and 10 DAP for the following comparisons: Midas versus G12873, AD versus AW; AD_Snap versus AD; AD_Snap versus AW; and MD versus MW.

### qRT–PCR of candidate genes for the *qPD5.1-Pv* locus

The indehiscent variety Midas and three parental lines of the IL mapping population with the highest level of pod shattering (ILs 232B, 244A/1A, and 038B/2A2), and that were near isogenic to Midas after three backcrosses, were grown in a greenhouse at the Max Planck Institute of Molecular Plant Physiology (Golm-Potsdam, Germany), in April to July 2018. The plants were individually grown in 20 cm diameter pots (volume, 3 litres), and fertilization was performed with Hakaphos rot (0.015%) during irrigation (666 g 10 l^–1^). The plants were watered four times per day, and pest control was performed using Neem Azal (6 ml 3 l^–1^). At least nine biological replicates were grown for each genotype. At least three pods from each dehiscent genotype and four pods for Midas were collected from different replicates, at 2, 3, 4, 5, 7, 9, 11, and 13 DAP. Entire green pods were collected from 2 DAP to 5 DAP, while from 7 DAP, the ventral and dorsal sutures were separated manually from the valves and collected separately to evaluate gene expression in the region surrounding the ventral suture. The pods were frozen in liquid nitrogen before storage at –80 °C. The pod tissues were ground with a mixer mill (MM400; Retsch), and the RNA was extracted using the RNA miniprep kit (Direct-zol; Zymo Research GmbH). The RNA was stained using GelRed, and its integrity was visualized using 1% agarose gels. The RNA concentrations and quality were measured using a spectrophotometer (NanoDrop OneC; Thermo Scientific). After adjusting the RNA concentrations, the cDNA was synthesized for each sample (Maxima First Strand cDNA Synthesis Kit with dsDNase; Thermo Scientific). Each cDNA was diluted 1:10, by adding HPLC quality water, and stored at –80 °C. The primers for the candidate genes (i.e. Real-time qRT–PCR) were designed based on the gene coding sequences using the Primer3 (v0.4.0) tool (Supplementary Table S5). The target candidate genes were selected based on gene annotation, gene expression from the RNA-seq data, the presence of selection signatures according to Schmutz *et al.* (2014) and Bellucci *et al.* (2014), the functions of orthologous genes, and the location in the genomic regions with high association with pod shattering. Two housekeeping genes were included, based on the literature [i.e. Phvul.007G270100 (Borges *et al.*, 2012) and Phvul.010G122200 (Montero-Tavera *et al.*, 2017)]. The amplification efficiencies were determined for each pair of primers. Here, four dilutions (i.e. 1:10, 1:20, 1:30, and 1:40) of the same cDNA were amplified (i.e. real-time qRT–PCR), and the slope (*R*^2^) of the calibration curve was used to infer the primer efficiency, according to Equation 1:

$$\begin{array}{l} Ef\;f\;iciency\;\left(\%\right)=\left(E1\right)\times 100 \end{array}$$ (1)

where E was obtained from *R*^2^ according to Equation 2:

$$\begin{array}{l} E=10^{1/slope} \end{array}$$ (2)

The differential gene expression was calculated as fold changes between each dehiscent line (i.e. 232B, 244A/1A, and 038B/2A2) and the indehiscent line Midas, and for all of the donor parents grouped together versus Midas, according to Schmittgen and Livak (2008). *t*-tests were performed for each comparison separately, as comparisons of the ΔCt values. ΔCt was obtained as the difference between the Ct (cycle threshold) of the candidate gene and the Ct of the housekeeping gene for normalization of gene expression, according to Schmittgen and Livak (2008).

### Identification of orthologous genes with putative functions in pod shattering

Here, we used the Orthofinder algorithm (Emms and Kelly, 2015) to identify clusters of orthologous genes among the proteomes of *P. vulgaris*, nine related legume species, and *A. thaliana*. The proteome sequences considered here were: *A. thaliana* (TAIR10); *P. vulgaris* (v2.1); *Glycine max* (L.) Merr. (Wm82.a2.v1); *Medicago truncatula* (G.) (285_Mt4.0v1); *Vigna unguiculata* (L.) Walp (v1.1); *Cicer arietinum* (L.) (cicar.I CC4958.gnm2.ann1); *Lotus japonicus* (L.) (v3.0); *Lupinus angustifolius* (L.) (1.0); *Vigna angularis* (W.) Ohwi & Ohashi (vigan.Gyeongwon.gnm3.ann1.3Nz5); *Vigna radiata* (L.) Wilczek (vigra.VC1973A.gnm6.ann1); and *Glycyrrhiza uralensis* (F.) (Gur.draft-genome.20151208). These were downloaded from: Phytozome (http://phytozome.jgi.doe.gov/); the ILS database (https://legumeinfo.org/); the *Lotus japonicus* genome assembly (http://www.kazusa.or.jp/lotus/); and the *Glycyrrhiza uralensis* genome database (http://ngs-data-archive.psc.riken.jp/Gur-genome/download.pl; Mochida *et al.*, 2017). The protein sequences from the primary transcripts were used for the analysis, except for *L. japonicus*, for which only the full proteome was available. Orthofinder (v2.1.2) identified 20 692 orthogroups (i.e. clusters of orthologous genes) across these 11 species. The list of structural genes involved in the synthesis of phenylpropanoid was obtained from the Plant Metabolic Network database (https://www.plantcyc.org/) for common bean, soybean, and *A. thaliana*, as pod shattering in common bean is positively associated with lignin content in the pods (Murgia *et al.*, 2017). Common bean genes without any clear annotation were considered as putative structural genes of phenylpropanoid synthesis if they clustered in the same orthogroup of *A. thaliana* and soybean lignin biosynthesis-related genes. *Arabidopsis thaliana* and soybean lignin-related genes that were not assigned to any orthogroup were blasted (BLASTp) against the common bean proteome to identify the best putative orthologues. Common bean gene orthologues to those with a well-known role or a putative function in seed dispersal mechanisms in *A. thaliana* and in other crops were also identified with the same approach.

### Identification of selection signatures

Genes that underwent selection during domestication of common bean in Mesoamerica and in the Andes (Schmutz *et al.*, 2014) were identified. Moreover, 27 243 contigs that were previously detected by Bellucci *et al.* (2014), which included 2364 putatively under selection in the Mesoamerican pool, were mapped against the last common bean genome version. The contigs were aligned against the *P. vulgaris* protein sequences of all of the gene coding sequences (annotation on Phytozome, version 2.1) using NCBI blastx (blast-2.2.26), and then the best hit for each contig was selected and the reference gene of each contig was established with a threshold of <1E-10. A gene was considered as putatively under selection if at least one of the five contigs with the best e-values was putatively under selection in Bellucci *et al.* (2014).

### Pod histological analysis on parental lines of the introgression line population

Pods of the highly shattering genotypes 232B, 244A/1A, and 038B/2A2 (ILs) and the totally indehiscent variety Midas were collected for histological investigation. These were from the same greenhouse experiment that was performed for the qRT–PCR expression analysis. In addition, replicates of genotype MG38 (RIL) were grown in the same experiment. Entire pods were collected across five developmental stages (6, 10, 14, 18, and 30 DAP). Then 2–3 cm free-hand cross-sections from the pods were fixed in 5% agarose, and 70, 50, and 30 µm cross-sections were obtained using a microtome (VT 1000 S; Leica). A solution of phloroglucinol (7 mg), methanol (7 ml), and 37% chloridric acid (7 ml) was applied to the pod sections for specific lignin staining. The pod sections were visualized under an optical microscope (BX51TF; Olympus).

## Results

### Histological modifications underlying pod shattering in common bean

**Fig. 1.**
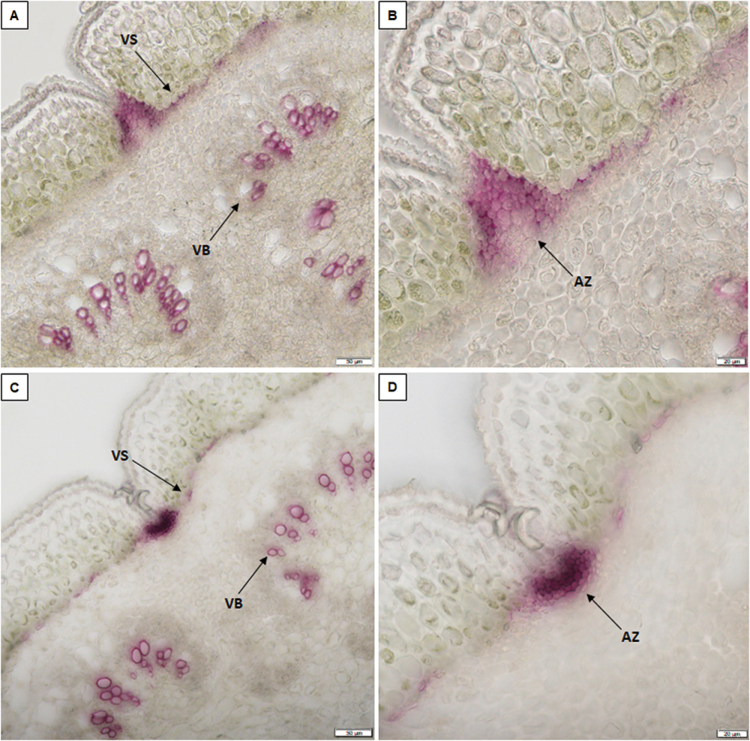
Analysis of lignification patterns in the ventral sheaths of 6-day-old pods of the totally indehiscent variety Midas and the highly dehiscent IL 244A/1A. Cross-sections (section thickness, 30 µm) of pods of Midas (A, B) and 244A/1A (C, D) after phloroglucinol staining for lignin. (B, D) Increased magnification from (A, C). Scale bars: 50 µm (A, C); 20 µm (B, D). VS, ventral sheath; VB, vascular bundles; AZ, abscission zone.

Lignification of the ventral and dorsal sheaths starts at 6 DAP for the pods of both the totally indehiscent variety Midas (Fig. 1A, B; Supplementary Fig. S3a, b) and the highly shattering IL 244A/1A (Fig. 1C, D; Supplementary Fig. S3c, d).

**Fig. 2.**
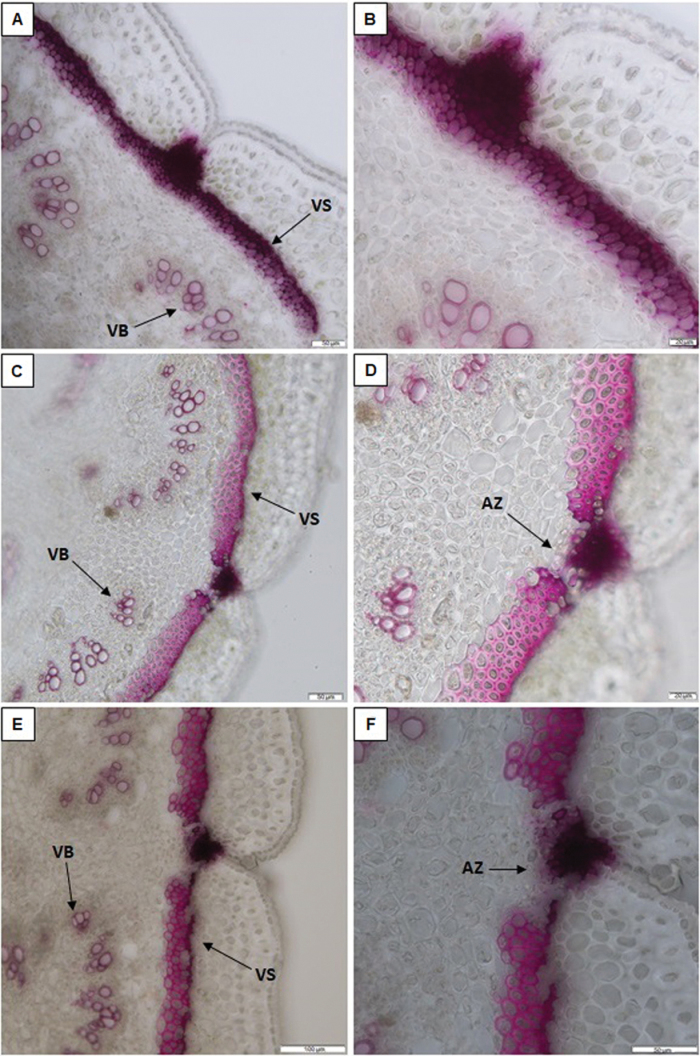
Analysis of lignification patterns in the ventral sheaths of 10-day-old pods of the totally indehiscent variety Midas and the highly dehiscent RIL MG38 and IL 244A/1A. Cross-sections (section thickness, 30 µm) of pods of Midas (A, B), MG38 (C, D), and 244A/1A (E, F) after phloroglucinol staining for lignin. (B, D, F) Increased magnification from (A, C, E). Scale bars: 50 µm (A, C, F); 20 µm (B, D); 100 µm (E). VS, ventral sheath; VB, vascular bundles; AZ, abscission zone.

Higher lignification was seen here for both the ventral (Fig. 1C, D) and the dorsal (Supplementary Fig. S3c, d) sheaths of the highly shattering IL 244A/1A, compared with the corresponding tissues of the indehiscent genotype Midas (Fig. 1A, B; Supplementary Fig. S3a, b). Moreover, a different conformation of the ventral sheath was seen comparing these non-shattering and highly shattering pods. For 10-day-old pods (i.e. at 10 DAP), the lignification pattern of the ventral suture clearly differed between the totally indehiscent variety (Fig. 2A, B) and the highly dehiscent RIL MG38 (Fig. 2C, D) and IL 244A/1A (Fig. 2E, F).

**Fig. 3.**
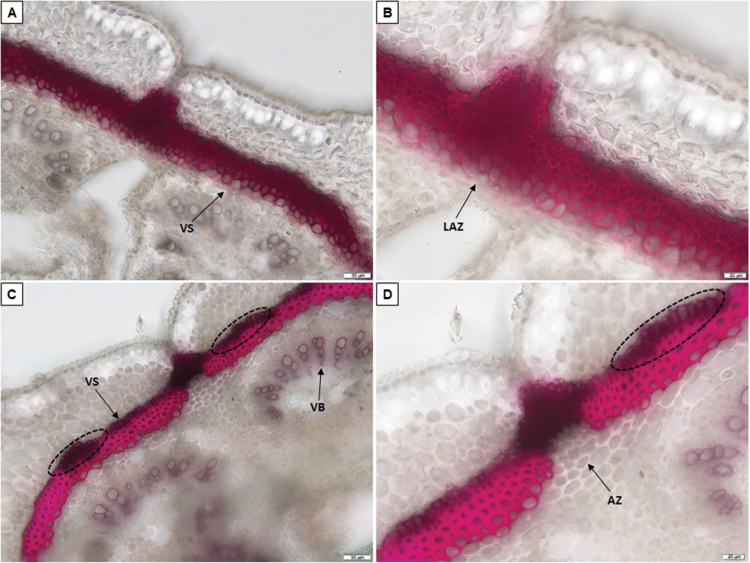
Analysis of lignification patterns of the ventral sheaths in 30-day-old pods (i.e. mature pods) of the totally indehiscent variety Midas and the highly dehiscent IL 038A/2A2. Cross-sections (section thickness, 50 µm) of the ventral suture of Midas (A, B) and 038A/2A2 (C, D) after phloroglucinol staining for lignin. (B, D) Increased magnification from (A, C). Scale bars: 50 µm (A, C); 20 µm (B, D). VS, ventral sheath; VB, vascular bundles; AZ, abscission zone; LAZ, lignified abscission zone. (C, D) Dotted ellipses, lignification areas with no strong cell wall thickening along the ventral sheath.

A few layers of cells were lignified in the abscission zone of the non-shattering type (Fig. 2B) compared with the equivalent tissue of the highly shattering lines (Fig. 2D, F), which lacked lignification. This modification is potentially involved in prevention of pod opening. The walls of the cells that surrounded the abscission zone in the ventral sheath were heavily thickened in the highly shattering pods (Fig. 2D, F), compared with the equivalent cells of the totally indehiscent pods (Fig. 2B). This might increase the mechanical tension within the ventral suture, to thus promote pod shattering. Moreover, at 10 DAP, the highly shattering pods showed an internal lignified pod valve layer (Supplementary Fig. S4b, c), which was not seen for the indehiscent pods of the variety Midas (Supplementary Fig. S4a). At 14 DAP, the degree of lignification of the ventral suture and both the ventral sheath and the abscission zone conformations strongly differed between the indehiscent variety Midas (Supplementary Fig. S5a, b) and the highly shattering RIL MG38 (Supplementary Fig. S5c, d) and IL 244A/1A (Supplementary Fig. S5e, f). The histological conformation of mature pods at 30 DAP is presented in Fig. 3.

In the region where the pods open at maturity (i.e. the abscission zone), in the highly shattering type, there were a few layers of cells that completely lacked lignification of the cell walls (Fig. 3D), compared with the lignification of the equivalent cells for the totally indehiscent pods (Fig. 3B). We therefore suggest that the non-functional abscission layer is responsible for the loss of pod shattering in common bean. The cell walls were heavily thickened in the ventral sheath of the highly shattering pods (Fig. 3D), compared with those of the ventral sheath of the indehiscent pods (Fig. 3B). The lumen of the cells also appeared to be almost occluded in some of the cells of the highly shattering pod sheaths. Interestingly, there were a few layers of lignified, but not heavily thickened, cells across the ventral sheath of the mature dehiscent pods (Fig. 3C, D, dashed ellipses). It is possible that different degrees of wall thickening along the sutures is required to create the mechanical tension needed for pod shattering and/or pod twisting. A schematic representation of the pod anatomy, depicting the main tissues putatively involved in the pod shattering modulation, is presented in Supplementary Fig. S6.

### Segregation of pod shattering

Phenotyping for pod shattering on 100 lines from six BC_4_/F_1_ families revealed uniformity in F_1_ for the presence of pod shattering. Phenotyping of 509 BC_4_/F_2_ lines, from the first planting (i.e. the subset of lines that were sown on the 7 June 2016) and that uniformly reached maturation, identified 386 and 120 dehiscent (presence of pod shattering) and indehiscent (absence of pod shattering) plants, which fits the 3:1 expected ratio for a monogenic Mendelian trait (i.e. presence/absence of pod shattering) (χ ^2^=0.45) (Supplementary Table S1). The expected segregation ratio was also observed when each of the BC_4_/F_2_ subpopulations was analysed separately (Table 1). The expected phenotypic segregation ratio for a qualitative trait was also observed for a subset of lines from the BC_4_/F_3_ population (i.e. 62.5:37.5 dehiscent versus indehiscent) that produced enough pods for a reliable post-harvest phenotyping of pod shattering (356 putative dehiscent versus 193 putative indehiscent lines) (χ ^2^=1.29) (Table 2). Moreover, 354 BC_4_/F_2_ dehiscent ILs showed pod twisting to different degrees (classed as: 1% to <10%; ≥10% to <24%; ≥24%; Supplementary Table S1), while 32 dehiscent lines did not show any twisting; considering the epistatic effect of the major locus for pod indehiscence on chromosome Pv05 on additional loci for pod shattering (Rau *et al.*, 2019) and assuming the action of duplicated and independent genes with cumulative effects, this fits to a 15:1 twisting/non-twisting ratio (χ ^2^=2.74).

**Table 1.** Observed segregation for the trait of ‘pod shattering occurrence’ in the BC_4_/F_2_ population, and for each subpopulation

Midas cross	BC_4_/F_2_ population/subpopulation (*n*)		
	Total	Dehiscent	Indehiscent
× 232B	210	169	41
× 244A/1A	94	64	30
× 038B/2A2	44	29	15
× 038B/2C1	43	37	6
× 038A/2D1	78	56	22
× 038B/2B1	37	31	6
**Total**	**506**	**386**	**120**

**Table 2.** Results of the post-harvest phenotyping for pod shattering for 549 BC_4_/F_3_ ILs

Description	Indication	No. of lines
Pods that hardly open along the sutures	Putative indehiscent	193
Pods that can be opened along the sutures	Putative dehiscent	301
Extreme dehiscent pods (open easily, with snap/ twist under slight pressure)	Putative dehiscent	55

Due to the high correlation that was observed here between the field and post-harvest phenotyping of the BC_4_/F_2_ population (*r*=0.81; *P*=7.33×10^–118^), the post-harvest evaluation was also integrated into the subsequent analysis. In total, 1197 BC_4_/F_4_ ILs were phenotyped for pod shattering in the field and/or after harvesting. When the field and post-harvest phenotypes were combined [i.e. defined as the ‘SH y/n’ (pod shattering, yes/no) trait], 940 and 243 ILs were classified as dehiscent and indehiscent, respectively, while 11 ILs were classified as intermediate (Table 3). The ‘intermediate’ phenotype was assigned to those lines that did not show a clear dehiscent or indehiscent phenotype after combining information from two phenotypic evaluations (i.e. field and post-harvest). Overall, 721 F_3_ families were represented at the beginning of the BC_4_/F_4_ field experiment, from which 502 F_3_ families produced BC_4_/F_4_ progenies. Of these, 95 indehiscent F_3_ lines gave complete indehiscent F_4_ progeny, while segregation was still observed within 55 F_3_ families.

**Table 3.** Results of field and post-harvest phenotyping for pod shattering for 1197 BC_4_/F_4_ ILs

Phenotype score	Phenotype evaluation	Phenotype description	No. of BC_4_/F_4_ ILs	
			Effective	After filtering
0	Field	Plant with no shattered pods (indehiscent)	326	311
1	Field	Plant with at least one shattered pod (dehiscent)	866	859
**Total**	**Field**		**1192**	**1170**
0	Post-harvest	Extreme indehiscent pods which do not open along sutures (putative indehiscent)	52	51
1	Post-harvest	Pods that hardly open along the sutures (putative indehiscent)	181	179
1.5	Post-harvest	Intermediate phenotype between classes 1 and 2 (intermediate)	27	27
2	Post-harvest	Pods which can be opened along the sutures (putative dehiscent)	666	657
3	Post-harvest	Extreme dehiscent pods which open easily, producing a snap/twist when subjected to a slight pressure (high shattering)	266	266
**Total**	**Post-harvest**		**1192**	**1180**
0	Combined field+post-harvest (Sh y/n)	Indehiscent plant	243	238
0.5	Combined field+post-harvest (Sh y/n)	Intermediate plant	13	11
1	Combined field+post-harvest (Sh y/n)	Dehiscent plant	940	927
**Total**	**Combined field+post-harvest (Sh y/n)**		**1196**	**1176**

Field and post-harvest data were combined, and a new comprehensive score was adopted (Sh y/n) for the evaluation of pod shattering. For BC_4_/F_4_ ILs, the ‘effective’ numbers are those for which phenotypic data were acquired, and the ‘after filtering’ numbers are those for plants without 100% accurate data (e.g. few evaluable pods, disease).

### Genome-wide association study for pod shattering and fine mapping of the major locus *qPD5.1-Pv*

**Fig. 4.**
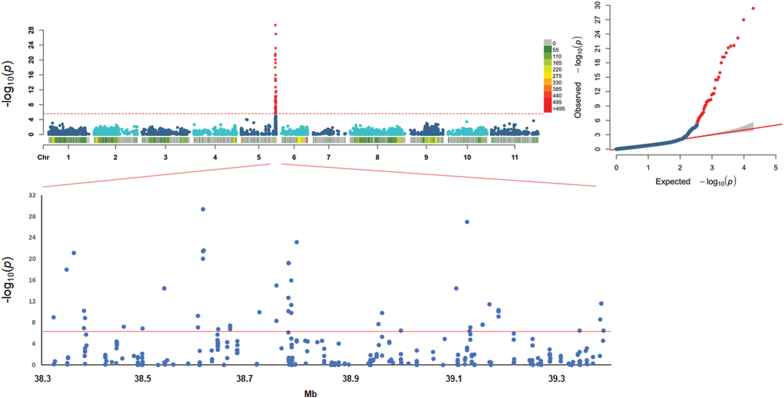
Genome-wide association study for occurrence of pod shattering. Top left: Manhattan plot to show the associations between 52 SNP markers (red dots on the distal region of chromosome Pv05) and the SH y/n trait (dehiscent versus indehiscent lines). Dashed red line, fixed threshold of significance for the 19 420 SNP markers physically distributed across the 11 common bean chromosomes. Top right: QQplot of the distribution of the observed *P*-values compared with the expected distribution. Bottom: expanded major QTLs on the distal part of chromosome Pv05, defining the significance of the SNP markers from 38.3 Mb to 39.4 Mb on chromosome Pv05.

A GWAS for pod shattering was performed using a dataset of 19 420 SNPs from GBS analysis, which were identified across 1196 BC_4_/F_4_ ILs (Supplementary Fig. S7). GWAS for the trait defined as ‘SH y/n’ (dehiscent versus indehiscent lines) identified a major locus for occurrence of pod shattering at the end of chromosome 5 (*qPD5.1-Pv*) (Fig. 4); here, 52 SNPs showed association (–log_10_*P*>6) with the presence/absence of pod shattering in the interval between the S5_38322754 and S5_39384267 markers.

The major locus *qPD5.1-Pv* was also in the association for the following mapping analyses: when 18 ILs for which the phenotype score was not clearly assigned were removed (see Table 3) (Supplementary Fig. S8a); when the ‘SH y/n’ trait that included plants with an intermediate phenotype was used (Supplementary Fig. S8b); when the presence/absence of pod shattering was only from the field phenotyping (Supplementary Fig. S8c); when the post-harvest phenotype was used (putative dehiscent versus putative indehiscent lines; Supplementary Fig. S8d); when all of the phenotypic classes from the post-harvest evaluation were used (quantitative score; Supplementary Fig. S8e); and when the percentage of twisting pods per plant was used (field evaluation; Supplementary Fig. S8f). These GWAS data are summarized in Table 4, while Supplementary Fig. S9 shows the expanded major QTLs for all of these mapping strategies. Supplementary Fig. S10 shows the slight decay of the linkage disequilibrium (LD) within the major QTL *qPD5.1-Pv* for pod indehiscence that does not exclude the presence of additional genes involved in pod shattering modulation within this region (average LD in the region; *r*^2^=0.47). Here, a few recurrent highly associated SNPs were identified within the major locus (Fig. 4; Supplementary Fig. S9; Table 4). These identified three genomic regions around 38.61, 38.79, and 39.12 Mb on chromosome Pv05. In particular, S5_38611412 was among the best associated SNPs for all of the mappings, with a few surrounding SNPs with high *P*-values (Table 4; Supplementary Fig. S9). After narrowing the QTL to a 22.5 kb surrounding region (from S5_38605293 to S5_38627793), a few candidates were identified, among which there was a protein kinase (Phvul.005G157300), a phospholipid-transporting ATPase (Phvul.005G157400; with the highest associated SNP S5_38611412), and a nucleotidase (Phvul.005G157500). The main peak was located ~11 kb before a MYB26 transcription factor (Phvul.005G157600), the orthologue that is involved in anther dehiscence and secondary cell wall differentiation in *A. thaliana* (Yang *et al.*, 2007). Moreover, a cluster of lipoxygenase genes were located on a tightly associated genomic region, from ~48 kb to ~17 kb upstream of the main peak (Phvul.005G156700, Phvul.005G156800, Phvul.005G156900, and Phvul.005G157000).

**Table 4.** Summary of the genome-wide association study for pod shattering in the BC_4_/F_4_ IL population

Phenotyping approach	Shattering trait	Sample size (no. of ILs)	Ch	Associated SNPs (*n*)	Genomic region	Three best-associated SNPs
**Shattering occurrence**						
Sh y/n	Dehiscent versus indehiscent	1183	Pv 05	52	38322754–39384267	S5_38611412 (4.35E-30) S5_38792327 (6.71E-24) S5_39120955 (1.03E-27)
Sh y/n	Dehiscent versus indehiscent (only accurate phenotypic scores)	1165	Pv 05	54	38322754–39384267	S5_38611412 (3.21E-27) S5_38792327 (5.06E-25) S5_39120955 (2.7E-27)
Sh y/n	All classes (dehiscent, intermediate, indehiscent)	1196	Pv 05	52	38322754–39384267	S5_38611412 (1.32E-31) S5_38792327 (5.77E-25) S5_39120955 (5.04E-27)
Field	Dehiscent versus indehiscent	1192	Pv 05	38	38322754–39182106	S5_38611085 (9.10E-20) S5_38611412 (2.73E-23) S5_38611464 (5.34E-19)
Post-harvest	Putative dehiscent versus putative indehiscent	1165	Pv 05	43	38322754–39379952	S5_38611085 (2.82E-27) S5_38611412 (2.79E-30) S5_38612876 (1.23E-26)
**Shattering modulation**						
Post-harvest	Quantitative (all classes)	1192	Pv 05	20	38348010–39120955	S5_38611412 (1.05E-15) S5_38611464 (5.43E-14) S5_38612876 (5.27E-15)
Field	% Twisting pods/plant	1002	Pv 05	7	38611085–38792327	S5_38611412 (3.51E-10) S5_38612876 (4.63E-08) S5_38792327 (4.77E-08)

The full list of SNPs that were significantly associated in at least one of the GWAS mapping experiments is reported in Supplementary Data S2, along with information on the physically closest genes.

### Identification of candidate genes for pod shattering and gene expression analysis

The candidate genes were identified based on the annotation, the function of orthologues in legume species and *A. thaliana*, the differential expression analysis using RNA-seq data between wild and domesticated pods, the differential expression at the target candidate genes for the major locus *qPD5.1-Pv* (Real-time qRT–PCR) in a comparison of NILs, and the evidence of selection signatures from Schmutz *et al.* (2014) and Bellucci *et al.* (2014).

#### Candidate genes at the major locus qPD5.1-Pv

Overall, *qPD5.1-Pv* contains 128 genes, of which 29 were differentially expressed (from RNA-seq data), and 15 were under selection in the Mesoamerican gene pool, according to Schmutz *et al.* (2014) and/or Bellucci *et al.* (2014). Four genes were both differentially expressed and under selection (Supplementary Data S3).

Located ~11 kb downstream of the most significant peak, Phvul.005G157600 is orthologous to *AtMYB26* (Yang *et al.*, 2007). Phvul.005G157600 expression was up-regulated in 5-day-old dehiscent pods (i.e. Midas versus G12873), and down-regulated in G12873 dehiscent pods at 10 DAP (Supplementary Data S3; row 50). Down-regulation of *PvMYB26* expression was also seen for the comparison of Mesoamerican domesticated and wild (MD versus MW) pods at 5 DAP (see also Supplementary Fig. S11 for expression data on the candidate genes). Moreover, two genes located downstream of *PvMYB26* (Phvul.005G157700 and Phvul.005G157800) on the physical map showed signatures of selection which might be due to ‘hitch-hiking’.

Within the highest associated region to which *qPD5.1-Pv* was narrowed down (S5_38605293:S5_38627793), Phvul.005G157400 and Phvul.005G157500 did not show differential expression or selection signatures, while no reads were mapped (i.e. RNA-seq) on Phvul.005G157300 in any of the samples (Supplementary Data S3; rows 47–49). In addition, *qPD5.1-Pv* contained a cluster of three differentially expressed linoleate 9S-lipoxygenase genes (Phvul.005G156700, Phvul.005G156900, and Phvul.005G157000; Supplementary Data S3; rows 41, 43, and 44) that were located upstream (from ~48 kb to ~17 kb) of the highest associated peak for pod indehiscence. Phvul.005G156700 was down-regulated for Midas versus G12873, and for Andean domesticated snap bean (AD_Snap) versus Andean wild (AW) at 10 DAP; Phvul.005G156900 expression was up-regulated for Midas versus G12873 at 10 DAP; while Phvul.005G157000 was down-regulated for the totally indehiscent pods (Midas versus G12873) at 10 DAP, and also showed signatures of selection in the Mesoamerican gene pool. In the region that surrounds SNP S5_39120955, which was also highly associated with the occurrence of pod shattering (see Table 4), there was a cluster of leucine-rich repeat (LRR) coding genes. In particular, Phvul.005G163800 and Phvul.005G163901 (Supplementary Data S3; rows 108 and 110) show differential expression for AD_Snap versus Andean domesticated dry beans (AD), AD versus AW, and MD versus MW at 5 DAP (Phvul.005G163800), and for Midas versus G12873 at 10 DAP (Phvul.005G163901). SNP S5_38792327 was also one of the best associated SNPs at the major locus *qPD5.1-Pv* (see Table 4), and it was located within a fatty acid omega-hydroxy dehydrogenase (Phvul.005G159400; Supplementary Data S3; row 69), which, however, did not show selection signatures or significant differential expression. Finally, Phvul.005G164800 showed higher expression in indehiscent pods of Midas at 5 DAP and 10 DAP, compared with G12873 (Supplementary Data S3; row 119), and it was annotated as ZINC FINGER FYVE DOMAIN-CONTAINING PROTEIN.

#### Candidate genes with a putative function in pod shattering based on their orthologues

Orthologous genes in common bean that in other species have pivotal roles in modulation of pod shattering, cell wall modifications, and putative pod-shattering-related functions were identified and are reported in Supplementary Data S4.

We consider as promising candidates the orthologous genes located close to the known QTLs for pod shattering. On chromosome Pv02, Phvul.002G271000 (*PvIND*; Gioia *et al.*, 2013) is orthologous to *AtIND* (Liljegren *et al.*, 2004), and it was highly expressed in the snap bean group compared with AW at 10 DAP (Supplementary Data S4; row 28); moreover, close to *PvIND*, we identified the NAC transcription factor Phvul.002G271700 (orthologous to NAC082). Both Phvul.002G271000 and Phvul.002G271700 map to the *St* locus (Koinange *et al.*, 1996). On chromosome Pv03, Phvul.003G252100 is orthologous to *PDH1* in soybean (Funatsuki *et al.*, 2014), which was recently proposed as a candidate for modulation of pod shattering in common bean (Parker *et al.*, 2020); here, Phvul.003G252100 was up-regulated for Midas versus G12873 at 5 DAP and 10 DAP, and down-regulated for AD versus AW at 5 DAP, and MD versus MW at 10 DAP, with a signature of selection in the Andean gene pool (Supplementary Data S4; row 45). On chromosome Pv04, Phvul.004G144900 is orthologous to the MYB52 transcription factor, which maps to a region associated with modulation of pod shattering (Rau *et al.*, 2019); here, Phvul.004G144900 was less expressed for AD_Snap versus AW and MD versus MW, both at 10 DAP (Supplementary Data S4; row 50). Moreover, ~660 kb downstream, Phvul.004G150600 is a PIN family member, and thus putatively involved in correct regulation of auxin efflux. Phvul.004G150600 showed higher expression for indehiscent pods (Midas versus G12873) at 5 DAP, with a signature of selection (Supplementary Data S4; row 51). On chromosome Pv09, close to the significant SNP for shattering modulation at ~30 Mb that was identified by Rau *et al.* (2019), and within the QTL identified also by Parker *et al.* (2020), Phvul.009G203400 is orthologous to *AtFUL* (Gu *et al.*, 1998); interestingly, Phvul.009G203400 shows parallel selection between the gene pools (Schmutz *et al.*, 2014), and congruently across different studies (Bellucci *et al.*, 2014; Schmutz *et al.*, 2014) (Supplementary Data S4; row 93). In the same region, two physically close genes, Phvul.009G205100 and Phvul.009G205200, are orthologous to *Cesa7*, and they showed selection signatures. Moreover, Phvul.009G205100 was less expressed in the domesticated pods (Supplementary Data S4; rows 94 and 95).

Here, we also identified potential candidates for pathways underlying pod shattering modulation at the genome-wide level based on their orthology with genes with well-described functions in the modulation of seed dispersal and/or fruit development in other species, and because they showed signatures of selection and/or interesting differential expression patterns. Those that can be highlighted are: Phvul.002G294800, as orthologous to *GmPDH1*; Phvul.003G166100 and Phvul.011G100300, as putatively orthologous to *Sh1*; Phvul.003G182700 and Phvul.003G281000, as orthologous to *AtFUL*; Phvul.007G100500, as putatively orthologous to *Shattering4*; Phvul.008G114300 and Phvul.010G011900, as orthologous to *Replumless*, *SH5*, and *qSH1*; and, in particular, Phvul.010G118700, as orthologous to *NST1* and *GmSHAT1-5* (Supplementary Data S4). These data suggest that these genes might share a conserved pod shattering-related function. Moreover, an *AtMYB26* orthologue on chromosome Pv10, Phvul.010G137500, was underexpressed in the AD and AD_Snap pods, compared with the wild pods at 5 DAP, while it was more highly expressed for MD versus MW at 10 DAP (Supplementary Data S4; row 100).

#### Structural genes in the phenylpropanoid biosynthesis pathway

In total, 109 genes were identified as putatively involved in the pathway of lignin biosynthesis based on gene annotation and orthologous relationships with genes from *G. max* and *A. thaliana* (Supplementary Data S5). No putative structural genes were identified within *qPD5.1-Pv*; however, several genes for lignin biosynthesis were located close to the major locus (Supplementary Fig. S12). According to the RNA-seq expression data here, 50 (46%) of the total 109 structural genes were significantly differentially expressed for Midas versus G12873, for at least one of the two developmental stages that were considered (*P*<0.01; with 41 of these at *P*<0.001) (Supplementary Data S5). This suggests that the developmental phase between 5 DAP and 10 DAP is of particular importance for pod lignin biosynthesis.

#### Expression patterns (Real-time qRT–PCR) of target candidates within the major locus qPD5.1-Pv

**Fig. 5.**
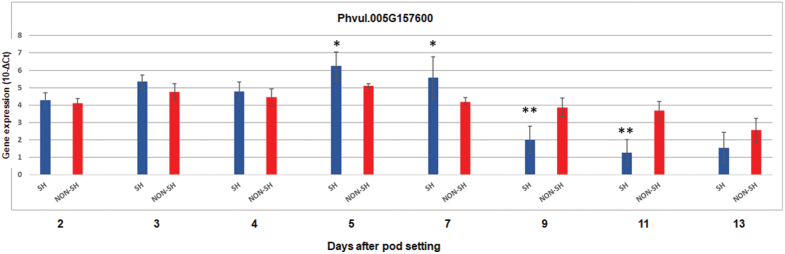
Gene expression by qRT–PCR for Phvul.005G157600 for the pods of the combined three highly dehiscent lines (SH; blue) and for the indehiscent pods of variety Midas (NON-SH; red) across the eight developmental stages from 2 DAP to 13 DAP. The mean pod expression for the three highly dehiscent introgression lines (038B/2A2, 244A/1A, 232B) is shown. *P<0.05; **P<0.01; SH versus NON-SH. Data are means ±SD of the biological replicates (*n*=3 for each highly dehiscent line for a total of nine for SH; *n*=4 for NON-SH). *t*-test for detection of significant differences, homoscedastic, two tails.

The expression pattern for *PvMYB26* (Phvul.005G157600) was investigated in the pods of the totally indehiscent variety Midas, as well as for the three near isogenic ILs 038B/2A2, 244A/1A and 232B across eight pod developmental stages, using Real-time qRT–PCR. Up to the 4 DAP stage, no differential expression was seen between the mean expression of the three highly shattering lines and the totally indehiscent Midas (Fig. 5).

*PvMYB26* (Phvul.005G157600) was up-regulated at 5 DAP and 7 DAP in the dehiscent pods (fold change, 2.20 and 2.62, respectively; Supplementary Table S6), although at 7 DAP, only the expression of IL 232B was significantly different from Midas (Supplementary Fig. S13). At 9 DAP, and with greater differences seen also at 11 DAP, *PvMYB26* was more highly expressed in the indehiscent pods of the variety Midas, as compared with the dehiscent lines, both as their combined mean expression (Fig. 5) and as their individual expression (Supplementary Fig. S13; Supplementary Table S6). Reassuringly, the expression patterns for *PvMYB26* (Phvul.005G157600) were in agreement between the RNA-seq data (Midas versus G12873; Supplementary Data S3; row 50) and the qRT–PCR data. Among the target candidates for the major locus, efficient amplification was obtained for: Phvul.005G156900 (linoleate 9S-lipoxygenase); Phvul.005G161600 (translation initiation factor 2 subunit 3); Phvul.005G161800 {rRNA [uracil(747)-C(5)]-methyltransferase}; Phvul.005G161900 (bHLH87 transcription factor similar to *AtIND*); Phvul.005G163901 (LEUCINE-RICH REPEAT PROTEIN KINASE-RELATED); Phvul.005G164800 (ZINC FINGER FYVE DOMAIN-CONTAINING PROTEIN); Phvul.005G165600 (auxin-responsive protein IAA18-related); Phvul.005G165900 (LYSM domain receptor-like kinase); and Phvul.005G166300 (Myb-like DNA-binding domain). Phvul.005G161900 showed overall lower expression across the pod developmental stages and plant genotypes (for both qRT–PCR and RNA-seq) when compared with the other target candidates. However, slightly, but significantly, increased expression was seen for the dehiscent pods at 5 DAP (Supplementary Table S6).

As mentioned above, Phvul.005G156900 is a promising target candidate due to its genomic position and expression pattern (i.e. RNA-seq data). However, differential expression was observed only at 7 DAP for each of the dehiscent lines individually, but with variable expression patterns across the three dehiscent lines (Supplementary Table S6). Phvul.005G161800 showed higher expression in the dehiscent pods across all of the pod stages, with the greatest fold change (3.273) seen for 11 DAP (Supplementary Table S6). These qRT–PCR data suggest that Phvul.005G161800 has a shattering-related function. The LRR-protein kinase-related gene Phvul.005G163901 was highly expressed in the dehiscent pods, with the most consistent differences seen at 4 DAP and 13 DAP (Supplementary Table S6). However, its expression pattern differed from that for the RNA-seq data (Midas versus G12873; Supplementary Data S3; row 110). This can potentially be explained by its expression being modulated after the expression of other genes involved in pod shattering, and its function is indeed worth further investigation.

When the shattering lines were considered as a combined group, Phvul.005G165900 showed lower expression in the highly shattering pods at 9, 11, and 13 DAP (Supplementary Table S6). Moreover, the Phvul.005G165900 expression pattern was in agreement with the RNA-seq expression data (Midas versus G12873 at 10 DAP; see Supplementary Data S3; row 130).

Overall, the best target candidate genes for *qPD5.1-Pv* are summarized in Table 5.

**Table 5.** Summary of the best candidate genes at the major locus *qPD5.1-Pv*, according to the expression data (i.e. RNA-seq and real-time qRT–PCR), the presence of a selection signature (Bellucci *et al.*, 2014; Schmutz *et al.*, 2014), and gene annotation of the common bean gene and its orthologues in *A. thaliana* and other crops

Gene (Phvul.005)	Location (Pv05:)	Description	Notes	Further notes
G156700	38553404–38557416	K15718: linoleate 9S-lipoxygenase (LOX1_5)	2	Tightly associated with the best associated SNP S5_38611412 for pod shattering occurrence (IL population mapping)
G156900	38579286–38583074	K15718: linoleate 9S-lipoxygenase (LOX1_5)	2	Tightly associated with the best associated SNP S5_38611412 for pod shattering occurrence (IL population mapping)
G157000	38584392–38587980	K15718: linoleate 9S-lipoxygenase (LOX1_5)	2, 4	Tightly associated with the best associated SNP S5_38611412 for pod shattering occurrence (IL population mapping)
G157600	38638487–38640559	PTHR10641:SF656: MYB DOMAIN PROTEIN 26	1, 2, 3	*A. thaliana* MYB26 orthologue, tightly associated with the best associated SNP S5_38611412 for pod shattering occurrence (IL population mapping)
G163800	39127757–39136117	LEUCINE-RICH REPEAT PROTEIN KINASE-RELATED	2	Tightly associated with the highly associated SNP for pod shattering occurrence SNP S5_39120955 (IL population mapping)
G163901	39140725–39145086	PTHR27003:SF105: LEUCINE-RICH REPEAT PROTEIN KINASE-RELATED	2, 3	Tightly associated with the highly associated SNP for pod shattering occurrence SNP S5_39120955 (IL population mapping)
G161900	38987320–38989140	PTHR12565:SF174: TRANSCRIPTION FACTOR BHLH87	3	Similar to *A. thaliana* ATIND (Indehiscent)
G161800	38972673–38978648	2.1.1.189: 23S rRNA [uracil(747)-C(5)]- methyltransferase	3	
G165900	39320080–39324951	LYSM DOMAIN RECEPTOR-LIKE KINASE	2, 3	
G157200	38600977–38601816	PF12023: Domain of unknown function (DUF3511) (DUF3511)	2, 4	
G164800	39245115–39246843	PTHR22835//PTHR22835:SF170: ZINC FINGER FYVE DOMAIN CONTAINING PROTEIN // SUBFAMILY NOT NAMED	2	

Target candidate for seed shattering based on:

Note 1: orthologues in *A. thaliana* and/or in other crop species have known or putative functions in the dehiscence processes, or have potentially related activities (e.g. cell wall modification, flower and fruit development).

Note 2: gene is differentially expressed (i.e. RNA-seq data), with an interesting expression pattern.

Note 3: Gene is differentially expressed (i.e. qRT–PCR) in the comparison of the near isogenic lines (three highly dehiscent introgression lines versus totally indehiscent Midas).

Note 4: presence of selection signature.

## Discussion

Our results confirm that pod indehiscence in snap beans is controlled by a Mendelian locus with recessive inheritance. Here, we narrowed the major QTL *qPD5.1-Pv* down to a 22.5 kb genomic region that is located ~11 kb upstream of *PvMYB26*. Among the candidate genes for loss of pod shattering, *PvMYB26* is the best candidate because of its specific differential expression pattern between dehiscent and indehiscent pods, which is in agreement with the histological modifications associated with pod shattering across the same pod developmental phases. Moreover, the histological modifications are consistent with the function of *AtMYB26* in *A. thaliana*. Here, we also provide a list of candidate genes potentially involved in pod shattering-related functions, through orthologue identification, selection signatures, and differential gene expression between wild and domesticated pods (i.e. RNA-seq) and/or between NILs (i.e. qRT–PCR).

We also demonstrate that pod indehiscence is associated with a lack of a functional abscission layer in the ventral sheath, due to ectopic lignification of a few layers of cells. Also, the key phenotypic events associated with pod shattering arise early in pod development, between 6 DAP and 10 DAP.

### Phenotypic architecture of pod shattering

Here, we propose that the failure of the formation of the abscission layer due to ectopic lignification is associated with pod indehiscence (see Fig. 3). This is similar to the ‘welding’ mechanisms previously defined for soybean by Dong *et al.* (2014), and more recently reported by Takahashi *et al.* (2019*a*) in an ethylmethane sulfonate (EMS) mutant of *Vigna stipulacea* Kuntze. Moreover, the cell wall thickening pattern that we observed in the cells surrounding the abscission zone of the pods (see Fig. 3) is in agreement with previous studies on *A. thaliana*, where in the wild type, lignification at the valve margin close to the abscission layer is required for silique shattering (Liljegren *et al.*, 2004). Interestingly, valve margin lignification is also associated with pod coiling in *M. truncatula* (Fourquin *et al.*, 2013). We have also confirmed that an internal lignified valve layer forms in highly dehiscent pods, compared with indehiscent pods, which occurs early, before 10 DAP (see Supplementary Fig. S4) (Murgia *et al.*, 2017).Interestingly, lignin deposition in the sclerenchyma of pod valves that is mediated by *GmPDH1* was associated with pod dehiscence modulation and pod twisting in soybean (Funatsuki *et al.*, 2014). This parallelism further reinforces the occurrence of convergent phenotypic evolution at the histological level between common bean and soybean for loss and reduction of pod shattering. Similarly, in some Brassicaceae, such as *Cardamine hirsuta* (L.), asymmetric lignin deposition in *endocarp b* of the silique valves also ensures explosive seed dispersal and silique coiling (Hofhuis *et al.*, 2016). Furthermore, we propose that the key histological modifications associated with pod shattering occur between 6 DAP and 10 DAP. This agrees with the observation that 46% of the putative structural genes of lignin biosynthesis are differentially expressed in the same phase when comparing indehiscent and highly shattering pods. Finally, from the phenotypic segregation analysis here (Supplementary Table S1), the modulation of pod twisting appears to be regulated in shattering pods by the action of at least two independent loci that are hypostatic to the major locus on chromosome Pv05.

### *PvMYB26*: the best candidate for the major locus *qPD5.1-Pv*

Among the candidate genes that we investigated, we propose *PvMYB26* as the best candidate at the major locus for pod indehiscence. This is based on its genomic location, on the parallel analysis of its expression patterns between dehiscent and indehiscent pods, and of the histological modifications associated with pod shattering in the early phase of pod development. A role for *PvMYB26* in the loss of pod shattering is strongly supported also by the function of its orthologue in *A. thaliana*. Indeed, *AtMYB26* is required to establish which cells undergo cell wall thickening to promote anther dehiscence (Yang *et al*., 2007, 2017), and it acts upstream of the *NST1* and *NST2* genes, which have key roles in silique shattering (Mitsuda and Ohme-Takagi, 2008). Interestingly, Takahashi *et al.* (2019*b*, Preprint) reported that pod shattering and pod tenderness are associated with MYB26 orthologues in azuki bean (*V. angularis*) and cowpea (*V. unguiculata*). The parallel identification of the MYB26 orthologue as the best candidate gene in *P. vulgaris* and other legumes (Takahashi *et al.*, 2019*b*, Preprint), in addition to previous data from Rau *et al.* (2019) and Lo *et al.* (2018) in common bean and cowpea, respectively, further reinforce the hypothesis of the occurrence of molecular convergent evolution for domestication of pod shattering. Here, we suggest that a fine and tissue-specific regulation of *PvMYB26* can be associated either with an ectopic lignification at the dehiscence zone in indehiscent pods or with the cell wall thickening that we observed in the ventral sheath of dehiscent pods, consistent with its expression pattern.

In addition to *PvMYB26*, we identified other genes that are worth highlighting. A cluster of four lipoxygenase genes were identified here, and their orthologues in *A. thaliana* (AT1G55020 and AT3G22400) are putatively involved in defence responses, jasmonic acid biosynthesis, and responses to abscisic and jasmonic acid [The Arabidopsis Information Resource (TAIR) database]. We also highlight Phvul.005G163901 and Phvul.005G163800 within a cluster of LRR genes, and Phvul.005G161800 {rRNA [uracil(747)-C(5)]-methyltransferase}. Interestingly, a potential role for LRR-RLK genes in shattering-related functions, such as secondary cell wall biosynthesis and abscission processes, can be postulated according to Jinn *et al.* (2000), Xu *et al.* (2008), Bryan *et al.* (2011), and Van Der Does *et al.* (2017). Finally, although Phvul.005G161900 (a bHLH87 transcription factor gene similar to *AtIND*) did not show a particular differential expression pattern between dehiscent and indehiscent pods, its involvement in the pod shattering modulation could not be excluded and its function is worth further investigation.

Overall, no putative structural genes for lignin fell within *qPD5.1-Pv* (see Supplementary Fig. S12). We suggest that selection might preferentially act on regulation factors instead of genes with a central role in the lignin biosynthetic pathway, perturbations of which can result in side effects on genotype fitness and/or can be disabling for normal development of the plant. However, the presence of putative structural genes for lignin biosynthesis close to *qPD5.1-Pv* suggests that they are directly involved in the same pathway as the genes responsible for the major QTL.

Based on the evidence we present here, *PvMYB26* is the best candidate for the major locus. Nevertheless, the presence of further candidates that are also organized within a cluster of genes leads to speculation that the main QTL operates in an ‘operon’-like manner. Indeed, the clustering of duplicated or non-orthologous genes might provide advantages in terms of coordination of expression between physically close genes that are involved in the same pathway, such as for secondary metabolite biosynthesis (Osbourn, 2010; Boycheva *et al.*, 2014).

### Convergent evolution and conservation of the molecular pathway for modulation of pod shattering

In the present study, we identified orthologous genes that are putatively involved in pod shattering-related functions (Supplementary Data S4). Among these, we highlight Phvul.002G271000 (*PvIND*), as orthologous to *AtIND* (Liljegren *et al.*, 2004), which has a pivotal role in silique shattering in *A. thaliana*. Moreover, our expression data and selection signatures reinforce the orthologue of *PDH1* (Funatsuki *et al.*, 2014) (*PvPdh1*; Phvul.003G252100) as a strong candidate for modulation of pod shattering also in common bean (Parker *et al.*, 2020). This might further suggest the occurrence of selection at orthologous loci for loss or reduction of pod shattering between closely related legume species (Supplementary Table S7). In addition, Phvul.009G203400 is a promising target candidate that shows parallel selection across the Andean and Mesoamerican gene pools, according to both Schmutz *et al.* (2014) and Bellucci *et al.* (2014). Moreover, Phvul.009G203400 is orthologous to *AtFUL* (Gu *et al.*, 1998) which is involved in valve differentiation in *A. thaliana*. Here, we also identified Phvul.010G118700 as orthologous to *NST1* (Mitsuda and Ohme-Takagi, 2008) and *GmSHAT1-5* (Dong *et al.*, 2014), which have crucial roles in silique shattering and in pod shattering resistance in *A. thaliana* and soybean, respectively. In addition to the major candidate *PvMYB26*, we also identified several MYB-like protein-coding genes close to known QTLs or at the genome-wide level (Supplementary Data S4), and, among these, a paralogue to *PvMYB26* on chromosome Pv10. The function of MYB transcription factors in the regulation of both secondary cell wall biosynthesis and the phenylpropanoid pathway has been widely reported (Zhong *et al.*, 2008; Zhang *et al.*, 2018). Overall, the expression patterns between the wild and domesticated pods, and the presence of selection signatures at orthologous genes at the genome-wide level (Supplementary Data S4), suggest that several of these have preserved shattering-related functions, and that there has been conservation across distant taxa of the pathway associated with seed dispersal mechanisms. This was previously demonstrated in rice (Konishi *et al.*, 2006; Yoon *et al.*, 2014), soybean (Dong *et al.*, 2014), and tomato (Vrebalov *et al.*, 2009).

## Supplementary data

The following supplementary data are available at *JXB* online.

Fig. S1. Schematic representation of the development of the BC_4_/F_4_ introgression line population.

Fig. S2. Structure of the GBS library.

Fig. S3. Analysis of lignification patterns in the dorsal sheaths of 6-day-old pods of the totally indehiscent variety Midas and the highly pod shattering IL 244A/1A.

Fig. S4. Analysis of lignification patterns in pod valves of 10-day-old pods of the totally indehiscent variety Midas and of the highly pod shattering RIL MG38 and IL 244A/1A.

Fig. S5. Analysis of lignification patterns in the ventral sheaths of 14-day-old pods of the totally indehiscent variety Midas and the highly pod shattering RIL MG38 and IL 244A/1A.

Fig. S6. Schematic representation of the pod anatomy, depicting the main tissues putatively involved in the pod shattering modulation.

Fig. S7. Densities of the 19 420 SNP markers identified within a 1 Mb window size using genotyping by sequencing.

Fig. S8. Genome-wide association study for occurrence of pod shattering in the IL population.

Fig. S9. Expanded major QTL for pod shattering on chromosome Pv05.

Fig. S10. Decay of the linkage disequilibrium within the major locus for pod indehiscence *qPD5.1-Pv*.

Fig. S11. Gene expression (RNA-seq) in common bean pods for candidate genes at the major locus for pod indehiscence.

Fig. S12. Physical positions of the putative structural genes for lignin biosynthesis on the common bean chromosomes.

Fig. S13. Gene expression by qRT–PCR for Phvul.005G157600 for the pods of the three highly dehiscent ILs and for the indehiscent pods of variety Midas across the eight developmental stages from 2 DAP to 13 DAP.

Table S1. Segregation of pod shattering on a subset of the BC_4_/F_2_ lines.

Table S2. Post-harvest phenotyping for the scoring of pod shattering of the IL population.

Table S3. Sequences of the single-stranded oligos for the adaptors used for GBS library preparation.

Table S4. Sequences of the primers used for the amplification, indexing, and quantification of the GBS library.

Table S5. Primer sequences for qRT–PCR and gene expression analysis of the target candidate genes at the major locus *qPD5.1-Pv* for pod indehiscence.

Table S6. Differential gene expression by qRT–PCR of the target candidate genes at the major locus *qPD5.1-Pv* for pod indehiscence.

Table S7. Orthologous genes putatively involved in pathways associated with pod shattering modulation across different species.

Dataset S1. List of accessions that were grown for pod collection, RNA-seq, and differential gene expression analyses.

Dataset S2. Significant SNPs identified across different GWAS mapping experiments at the major locus *qPD5.1-Pv* for loss of pod shattering.

Dataset S3. Genes identified within the major locus *qPD5.1-Pv* for loss of pod shattering.

Dataset S4. Genes in common bean that are orthologous to genes in other species with known functions that are putatively involved in seed shattering or have potentially related functions (e.g. cell wall modification, differentiation).

Dataset S5. Genes in common bean that are putatively involved in the phenylpropanoid biosynthesis pathway.

eraa553_suppl_Supplementary_Tables_and_Figures

eraa553_suppl_Supplementary_Datasets

## Data Availability

The data supporting the findings of this study are available from the corresponding author, Professor Roberto Papa, upon request.
