# Supplementary material for: Pod indehiscence in common bean is associated with the fine regulation of PvMYB26
Source: J Exp Bot. 2020 Nov 28;72(5):1617–33. doi: 10.1093/jxb/eraa553 (PMC7921299; doi:10.1093/jxb/eraa553)
Supplement: eraa553_suppl_Supplementary_Tables_and_Figures [file eraa553_suppl_supplementary_tables_and_figures.pdf]

SUPPLEMENTARY DATA

Supplementary figures

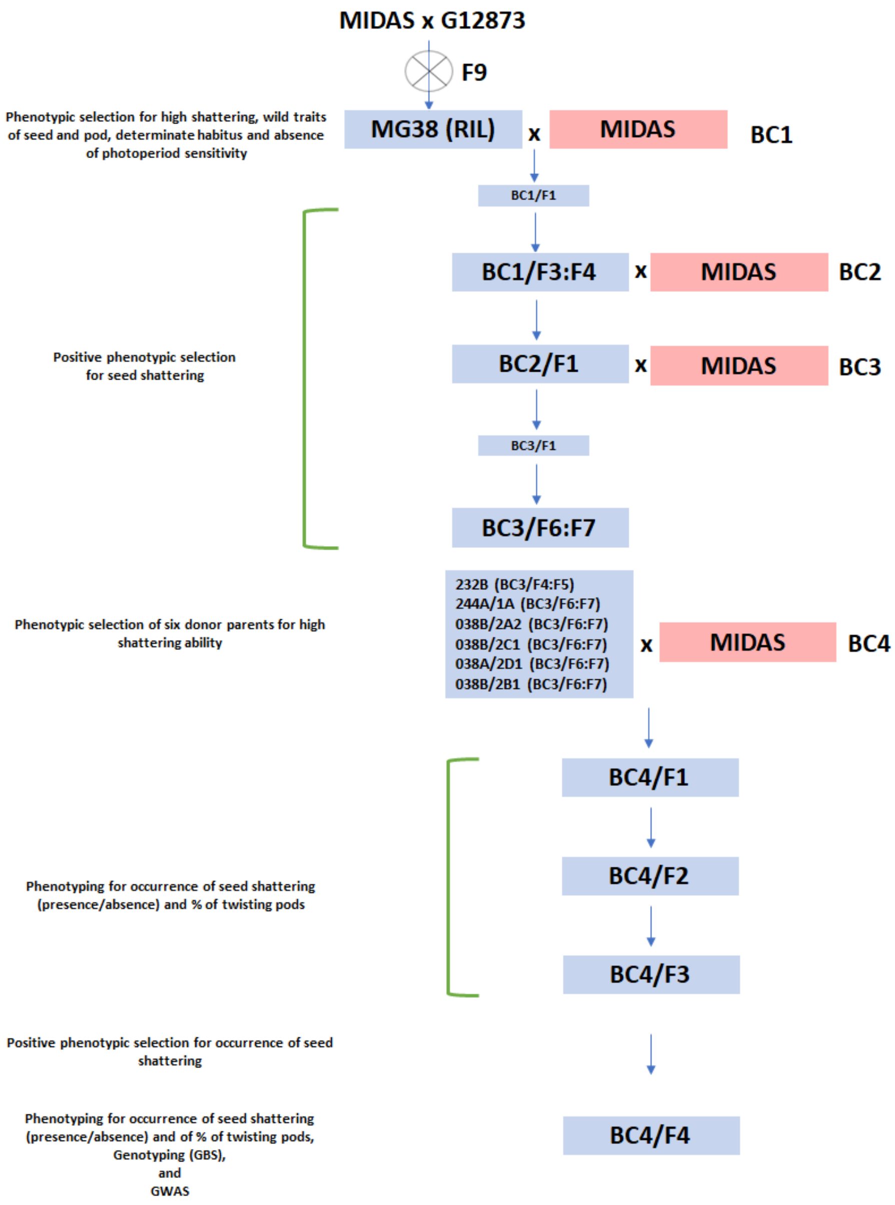

Figure S1. Schematic representation of the development of the BC4/F4 introgression line population. The initial cross was between the totally indehiscent Andean variety Midas and the highly shattering wild Mesoamerican genotype G12873, which provided a set of recombinant inbred lines. Then MG38 was selected as the donor parent for its high shattering ability, and further wild traits. MG38 was backcrossed with the recurrent parent Midas (BC1), and a strategy that combined self-pollination and two further backcrosses (BC2, BC3) with constant phenotypic selection for seed shattering provided ILs from the BC3/F4:F5 and BC3/F6:F7 families. Six lines that showed high shattering were selected as donor parental lines to be further backcrossed with the recurrent Midas (BC4). From the BC4/F3 population to the BC4/F4 generation, phenotypic selection for occurrence of seed shattering was performed, and only one seed was sown from each indehiscent F3 line, while at least 4 seeds (when available) were sown from each dehiscent F3 line.

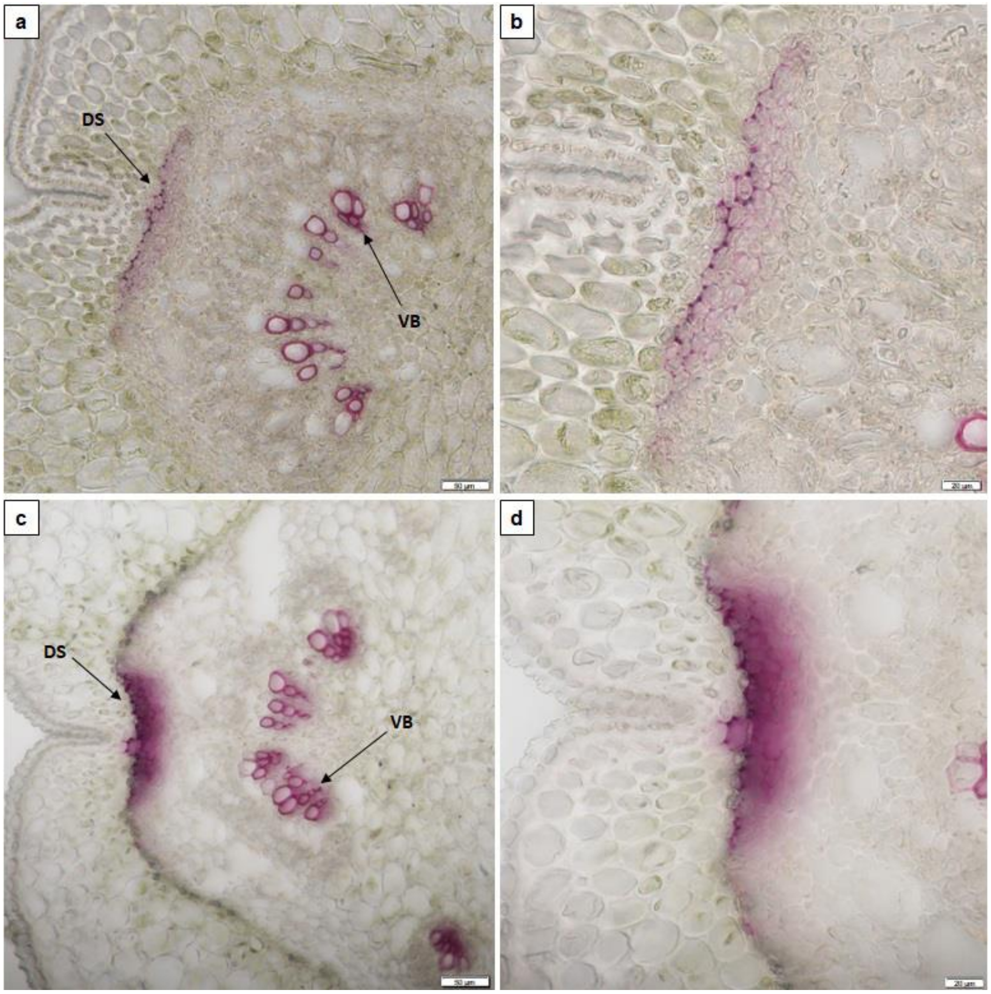

Figure S2. Analysis of lignification patterns in the dorsal sheaths of 6-day-old pods of the totally indehiscent variety Midas and the highly pod shattering IL 244A/1A. Cross-sections (section thickness, 30 µm) of pods of Midas (a, b) and 244A/1A (c, d) after phloroglucinol staining for lignin. (b, d) Increased magnification from (a, c). Scale bars: 50 µm (a, c); 20 µm (b, d). VB, vascular bundles; DS, dorsal sheath.

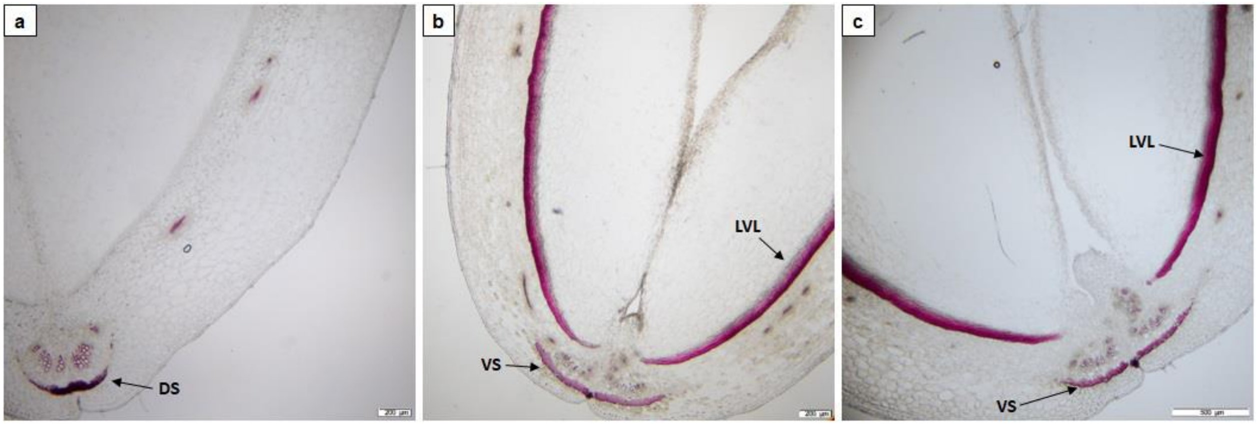

Figure S3. Analysis of lignification patterns in pod valves of 10-day-old pods of the totally indehiscent variety Midas and of the highly pod shattering RIL MG38 and IL 244A/1A. Cross-sections (section thickness, 30 µm) of pods of Midas (a), MG38 (b) and 244A/1A (c) after phloroglucinol staining for lignin. Scale bars: 200 µm (a, b); 500 µm (c). VS, ventral sheath; DS, dorsal sheath; LVL, internal lignified valve layer.

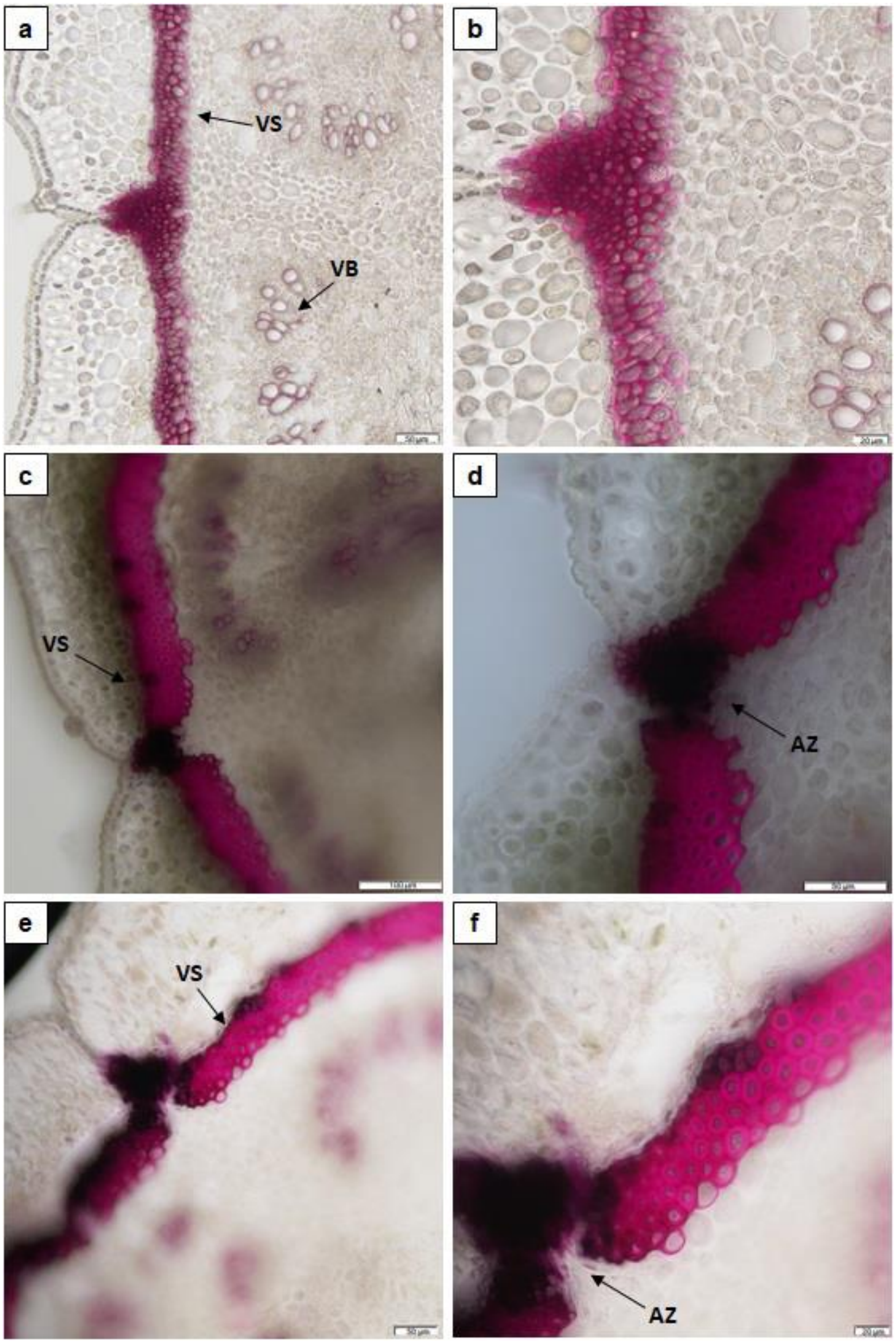

Figure S4. Analysis of lignification patterns in the ventral sheaths of 14-day-old pods of the totally indehiscent variety Midas and the highly pod shattering RIL MG38 and IL 244A/1A. Cross-sections (section thickness, 50 µm) of pods of Midas (a, b), MG38 (c, d) and 244A/1A (e, f) after phloroglucinol staining for lignin. (b, d, f) Increased magnification from (a, c, e). Scale bars: 50 µm (a, d, e); 20 µm (b, f); 100 µm (c). VS, ventral sheath; VB, vascular bundles; AZ, abscission zone.

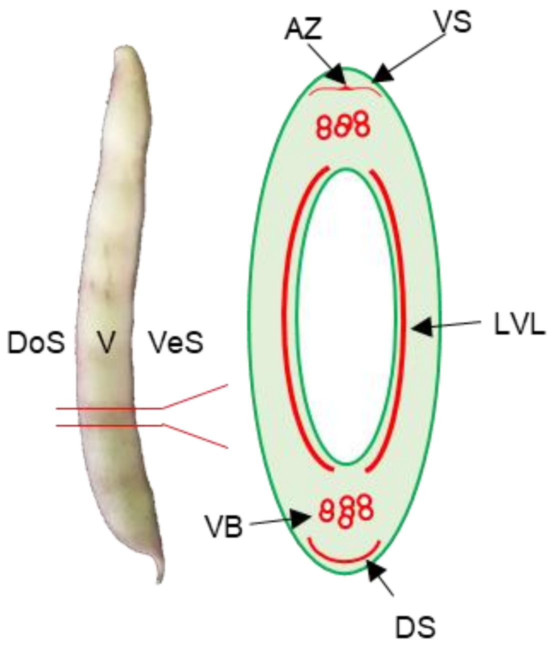

Figure S5. Schematic representation of the pod anatomy, depicting the main tissues putatively involved in the pod shattering modulation. Right, schematic representation of a cross-section of a pod in common bean. V, valve; DoS, dorsal suture; VeS, ventral suture; VS, ventral sheath; DS, dorsal sheath; AZ, abscission zone; LVL, internal lignified valve layer; VB, vascular bundles.

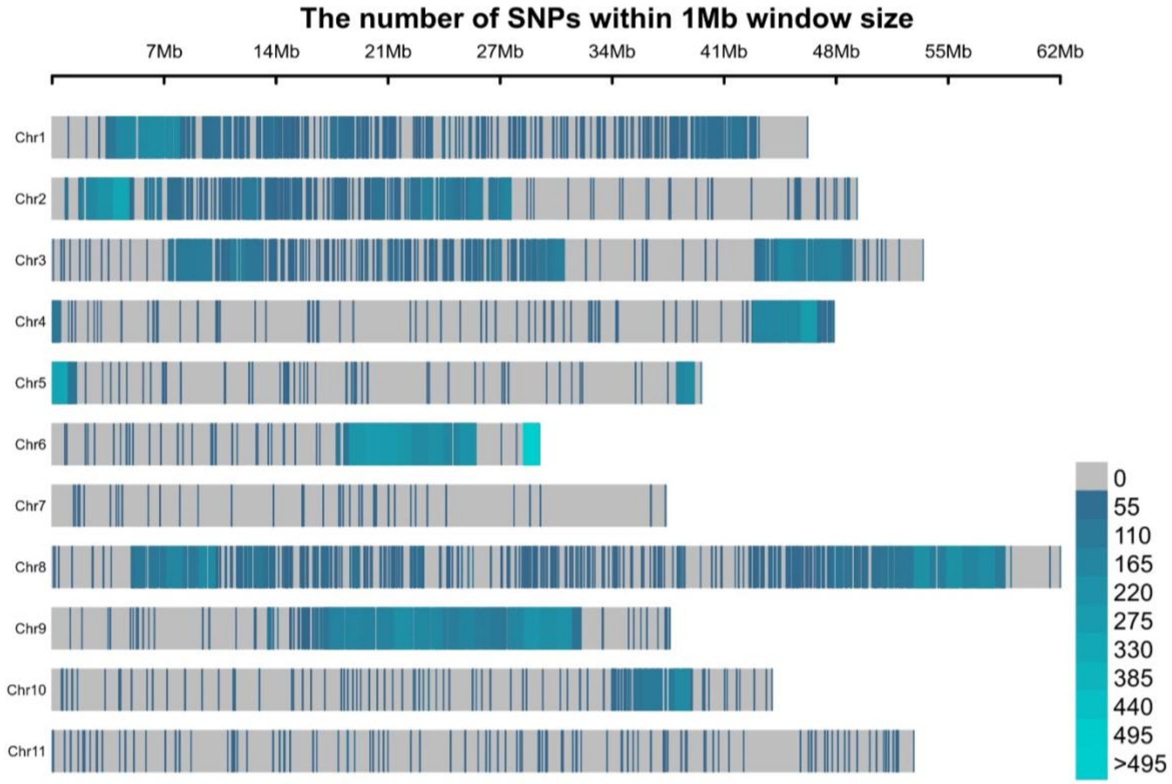

Figure S6. Densities of the 19,420 SNP markers identified within a 1-Mb window size using genotyping by sequencing.

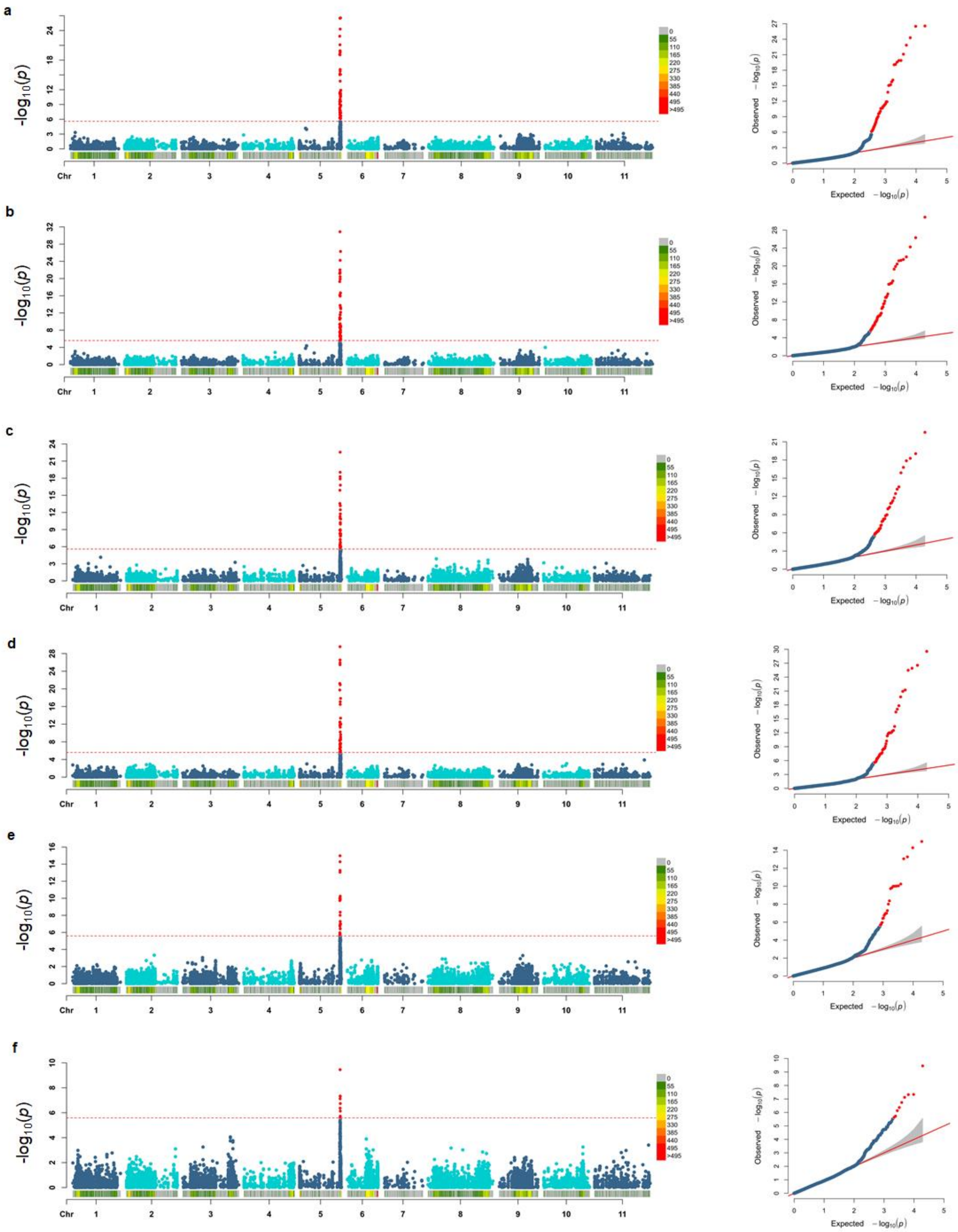

Figure S7. **Genome-wide association study for occurrence of pod shattering on the IL population**. Left: Manhattan plots to show the associations between the SNP markers and the phenotypic scores for pod shattering. Dashed red line, fixed threshold of significance for the 19,420 SNP markers physically distributed across the 11 common bean chromosomes. Right: QQplots of the distributions of the observed p values compared to the expected distribution. The following traits were mapped: (**a**) 'Sh y/n' (only clear phenotypic data, no intermediate phenotypes);

(**b**) ‘Sh y/n’ (intermediate phenotypes included); (**c**) Field (dehiscent *vs* indehiscent); (**d**) Post-harvest (putative dehiscent *vs* putative indehiscent); (**e**) Post-harvest (quantititative; mapping of phenotypic classes 0, 1, 1.5, 2, 3); (**f**) Proportion of twisting pods per plant (Field).

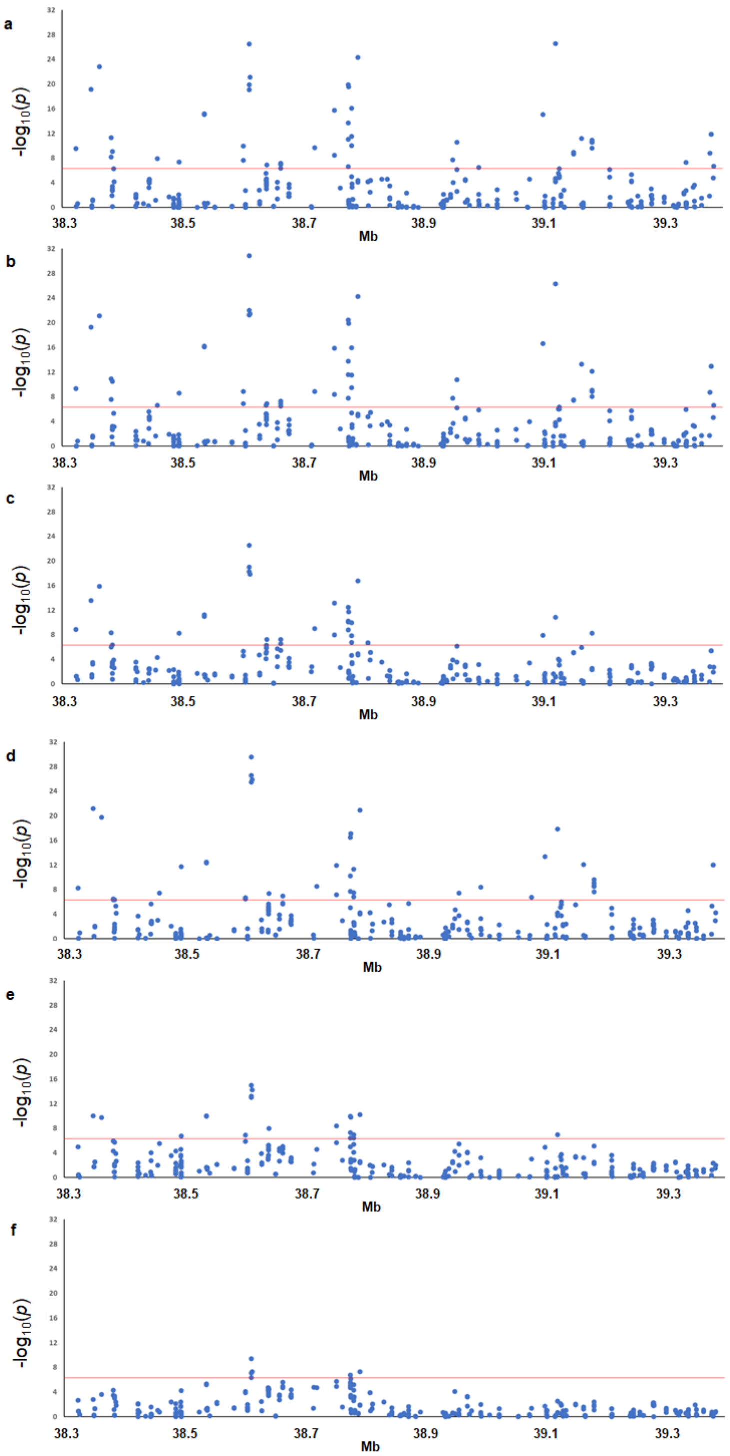

Figure S8. Expanded major QTL for pod shattering on chromosome Pv05. Dashed red line, fixed threshold of significance, defining the significance of the SNP positions from 38.3 to 39.4 Mb on chromosome Pv05. The SNPs were associated to the following traits: (**a**) ‘Sh y/n’ (only clear phenotypic data, no intermediate phenotypes; (**b**) ‘Sh y/n’ (intermediate phenotypes included); (**c**) Field (dehiscent *vs* indehiscent); (**d**) Post-harvest (putative dehiscent *vs* putative indehiscent); (**e**) Post-harvest (quantititative; mapping of phenotypic classes 0, 1, 1.5, 2, 3); (**f**) Proportion of twisting pods per plant (Field).

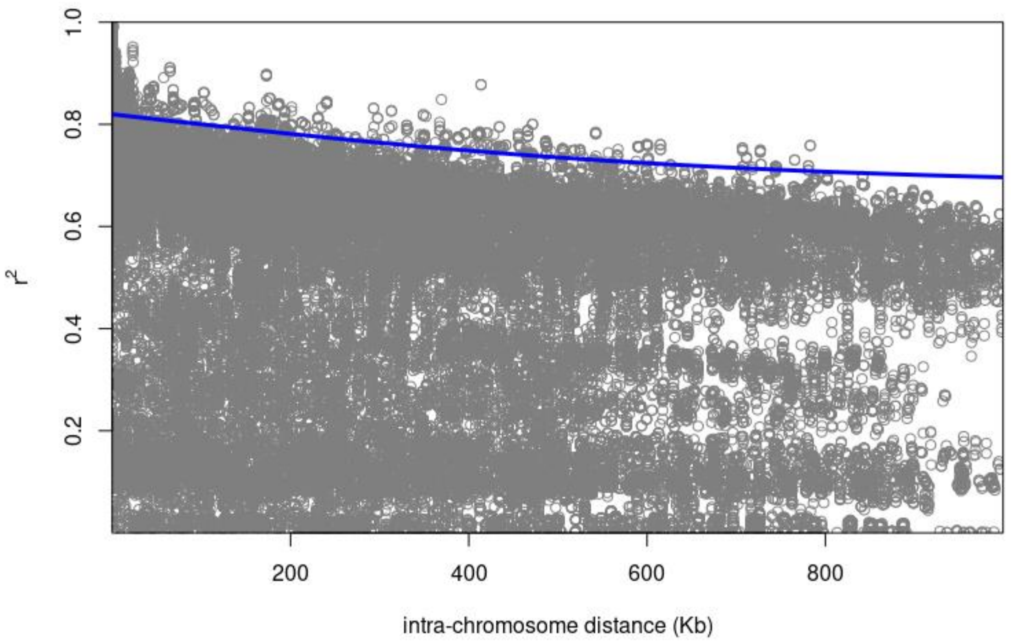

Figure S9. Decay of the linkage disequilibrium within the major locus for pod indehiscence *qPD5.1-Pv*. Intra-chromosome distance is expressed in Kilobases (Kb).

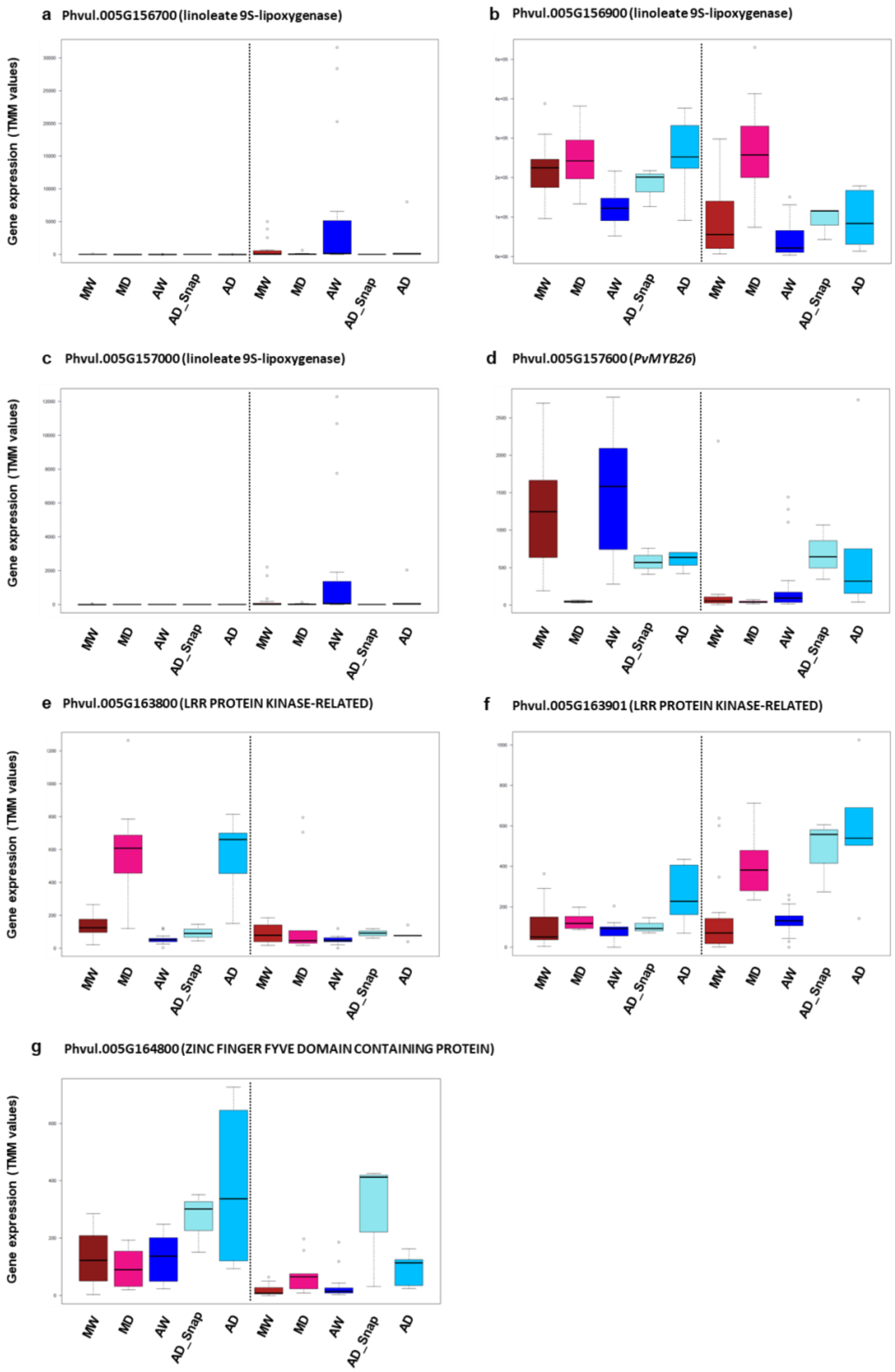

Figure S10. Gene expression (RNA-seq) in common bean pods for candidate genes at the major locus for pod indehiscence. Data are presented for five and ten old-days pods in a panel of wild and domesticated Mesoamerican and Andean genotypes. MW, Mesoamerican wild; MD, Mesoamerican domesticated; AW, Andean wild; AD_Snap, Andean snap beans (domesticated); AD, Andean dry beans (domesticated). Gene expression for candidate genes: a, Phvul.005G156700; b, Phvul.005G156900; c, Phvul.005G157000; d, Phvul.005G157600; e, Phvul.005G163800; f, Phvul.005G163901; g, Phvul.005G164800. The vertical dashed line separates the expression data for five old-days pods (left box) and for ten old-days pods (right box) in each panel. Data are means across TMM (Trimmed Means of M-values) ±standard deviation of the biological replicates.

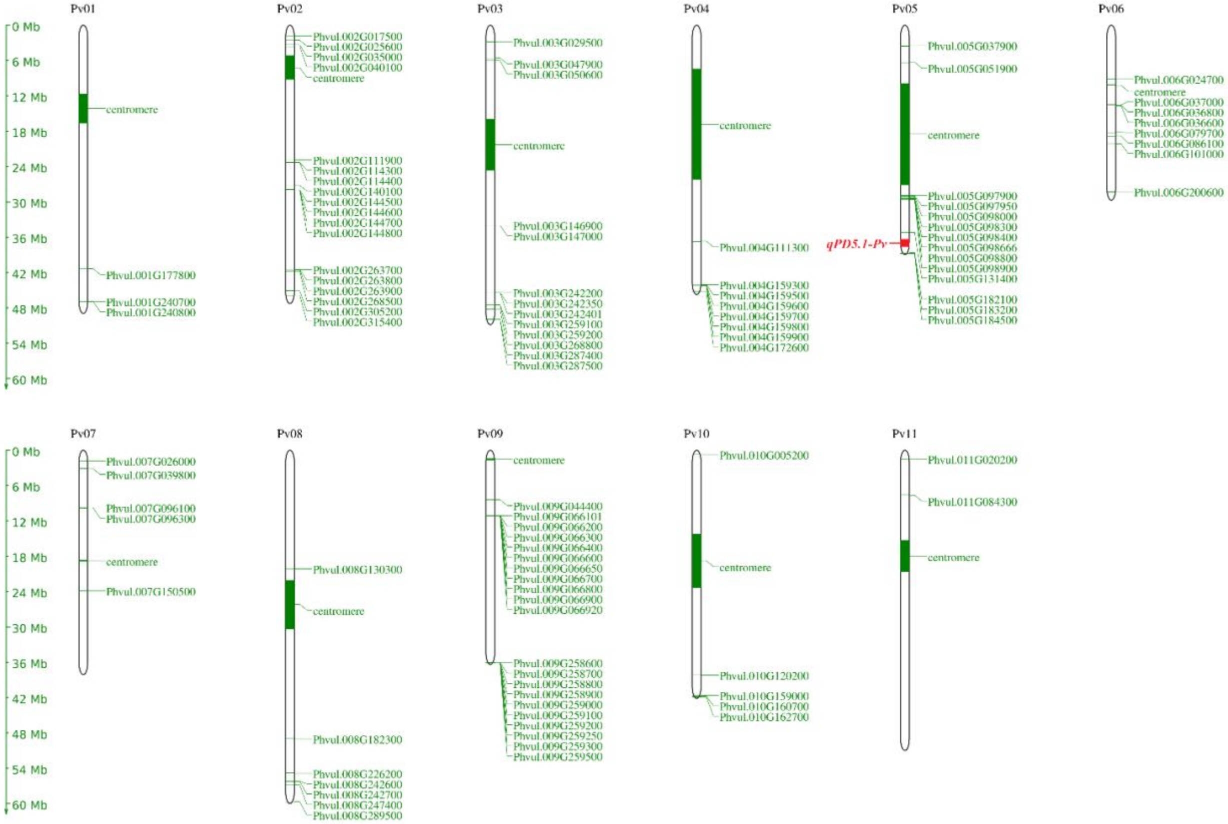

Figure S11. Physical positions of the putative structural genes for lignin biosynthesis on the common bean chromosomes. Red, left, position of the major locus *qPD5.1-Pv* on chromosome Pv05; green, right, genomic locations of the genes with putative functions in lignin biosynthesis. Centromeric regions are indicated for each of the chromosomes. Maps constructed using the online tool MapGene2Chrom Web2 (http://mg2c.iask.in/mg2c_v2.1/).

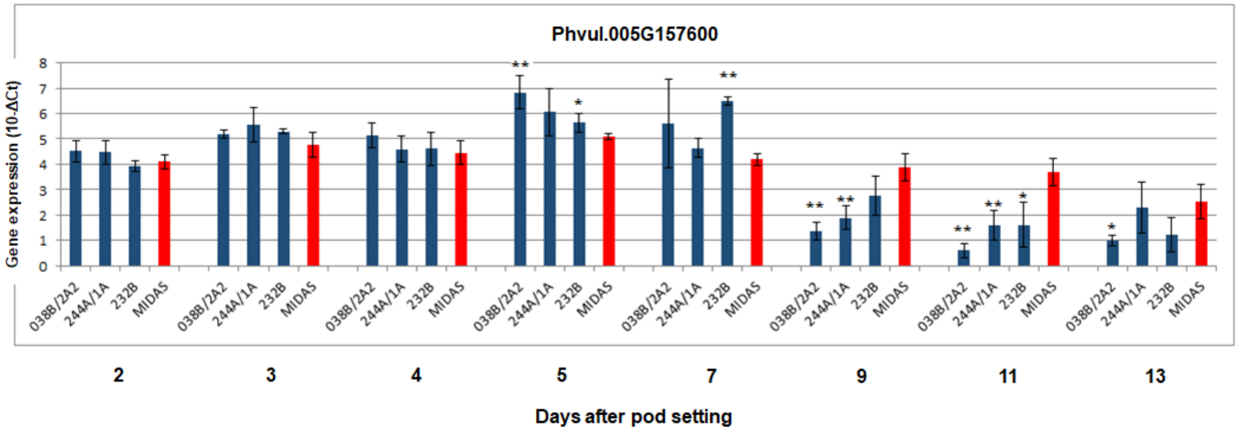

Figure S12. Gene expression by qRT-PCR for Phvul.005G157600 for the pods of the three highly dehiscent ILs (as indicated, blue) and for the indehiscent pods of variety Midas (MIDAS, red) across the eight developmental stages from 2 DAP to 13 DAP. Mean pod expression is shown. *, $p < 0.05$; **, $p < 0.01$; *versus* MIDAS. Data are means ±standard deviation of the biological replicates (n = 3 for each highly dehiscent line; n=4 for Midas). T.test for detection of significant differences, homoscedastic, two tails.

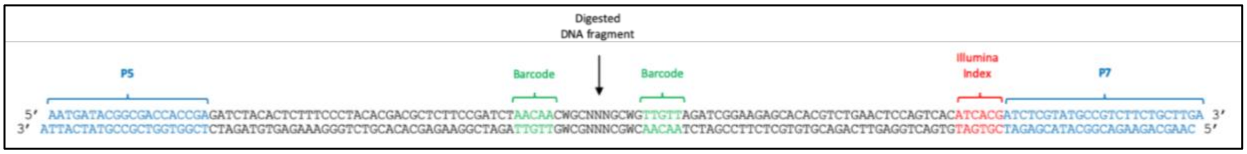

Figure S13. Structure of the GBS library. Samples-specific barcode, pool-specific Illumina index and P5/P7 annealing sites are indicated.

Supplementary tables

Table S1. Segregation of pod shattering on a subset of the BC4/F2 lines. Phenotyping was performed on four days (19, 29 September, 13, 23 [full ripening] October, 2016), for the identification of totally indehiscent plants and dehiscent plants. Shattering modulation (% of twisting pods per plant) was scored for the dehiscent lines.

Phenotyping date	Plant phenotypic class (n)			Pod shattering (n) relative to %twisted pods/dehiscent plant			
	Indehiscent	Dehiscent	Total	None	1% to <10%	≥10% to <24%	≥24%
19 September	265	244	**509**	71	166	7	0
29 September	176	330	**506**	59	228	20	23
13 October	127	379	**506**	44	263	37	35
23 October	120	386	**506**	32	272	42	40

Table S2. **Post-harvest phenotyping for the scoring of pod shattering of the IL population.**

Score	Phenotypic description
0	Extremely indehiscent pods that do not open along the sutures (extremely indehiscent plant)
1	Pods that hardly open along the sutures (putative indehiscent plant)
2	Pods that can be opened along the sutures (putative dehiscent plant)
3	Extremely dehiscent pods that open easily, with a snap when subjected to pressure (extremely dehiscent plant)

Table S3. Primers sequences for qRT-PCR and gene expression analysis of the target candidate genes at the major locus *qPD5.1-Pv* for pod indehiscence.

Gene	Primer sequence	
	Forward	Reverse
Phvul.005G156700	CCCTCACTGCTTTCTTAGGC	CGTCCCATTCAAAACGAAC
Phvul.005G156900	CATTTGGTTACTGGCAAAGG	GGGCAAGCGAGTTAATGTTC
Phvul.005G157000	ACATCCTGGGGGTGATAAAT	TGACGAATGGCTCAACAAC
Phvul.005G157600	CAACTTATGGACACGGTTGC	AGGTAGGTGCTTGGCTATCTG
Phvul.005G161600	GTGTGATGTGCCAGGGTTT	TCAGAGGTTTGTGGTTGTGG
Phvul.005G161800	AGAACGGGAAGCGATGTAA	TCCTCTCCAACGGATGTGT
Phvul.005G161900	CCAGAGGCAGTAGCACAAATG	TTCCACCAGGCACAATCTTC
Phvul.005G163901	ATGGCTAAAGGGAACTTACGAG	AAGAGTGGTAGTGAGGGTCCAG
Phvul.005G164600	GACAGAGGACAAGGTTTTGGA	TGGTCTGAACCGCAGAAA
Phvul.005G164800	TGGCAGCAGTGATTTTGTG	CAACCATTCTCGTGGAAACC
Phvul.005G164900	TACAGGAAAAGGGCGTGAA	TGCTGCTGAAATGATGTTGC
Phvul.005G165300	ACATGGTCATCCAAGGTGAA	GGCCCTGCTCAATCAAATA
Phvul.005G165600	GAAAGCAATCACGGGCTTA	ATTTCATGGCCGAGTCAAG
Phvul.005G165900	TTGTGGATGTGGCAAACCT	TTGAGGCAAAAATCTCGTCA
Phvul.005G166300	GACGTTCCAGCCTTTTTGAC	TGGCCTGATCCATGTTTTC
Phvul.007G270100†	AAGGTATTGGAGACGGCACAG	GTGAATGAAACCGAACACTTGG
Phvul.010G122200†	AGGAACCGGGAAAACACTTC	CCAACACCATCATCAAATCG

†, housekeeping genes

Table S4. Differential gene expression by qRT-PCR of the target candidate genes at the major locus *qPD5.1-Pv* for pod indehiscence. Comparisons as fold-changes (shattering/ indehiscent) between the pods of the three high shattering lines, 232B, 244A/1A and 038B/2A2 (both individually and combined), and the indehiscent variety Midas across the different developmental stages (DAP). The best candidate Phvul.005G157600 (orthologue to *AtMYB26*) is reported first, followed by the other target candidates according to genomic location. Significance: yellow shading, p ≤0.05; red shading, ≤0.01.

Gene (Phvul.005)	DAP	038B/2A2 vs Midas Fold-change	038B/2A2 vs Midas p	244A/1A vs Midas Fold-change	244A/1A vs Midas p	232B vs Midas Fold-change	232B vs Midas p	Combined vs Midas Fold-change	Combined vs Midas p
G157600	2	1.34	0.202	1.29	0.243	0.89	0.395	1.13	0.474
	3	1.34	0.226	1.74	0.168	1.45	0.130	1.50	0.057
	4	1.60	0.123	1.09	0.741	1.12	0.713	1.25	0.329
	5	3.31	0.003	1.95	0.088	1.44	0.043	2.20	0.021
	7	2.68	0.158	1.37	0.099	4.90	0.000	2.62	0.047
	9	0.18	0.001	0.25	0.004	0.47	0.075	0.28	0.001
	11	0.12	0.000	0.24	0.004	0.24	0.011	0.19	0.000
	13	0.34	0.013	0.85	0.719	0.40	0.089	0.50	0.076
G156900	3	1.79	0.011	1.17	0.600	1.45	0.100	1.45	0.072
	5	1.30	0.185	1.08	0.705	1.03	0.892	1.14	0.367
	7	0.52	0.036	0.46	0.046	2.68	0.044	0.86	0.768
	9	0.68	0.560	0.90	0.883	0.94	0.944	0.83	0.687
	11	1.02	0.986	1.22	0.866	0.88	0.899	1.03	0.965
	13	2.22	0.559	1.25	0.846	1.33	0.782	1.48	0.567
G161600	3	1.22	0.100	1.62	0.013	1.37	0.126	1.39	0.035
	5	0.79	0.061	0.93	0.490	1.00	0.996	0.89	0.197
	7	0.88	0.266	1.08	0.485	1.20	0.063	1.05	0.649
	9	1.08	0.455	1.34	0.035	1.27	0.030	1.22	0.029
	11	1.15	0.378	1.19	0.369	1.08	0.645	1.14	0.297
	13	1.29	0.106	1.49	0.016	1.24	0.152	1.34	0.022
G161800	3	2.99	0.002	2.24	0.006	2.16	0.008	2.44	0.000
	5	2.02	0.000	1.97	0.003	2.80	0.000	2.17	0.000
	7	1.48	0.182	1.55	0.112	2.31	0.006	1.74	0.014
	9	4.12	0.001	2.59	0.020	2.15	0.008	2.84	0.001
	11	3.94	0.000	3.32	0.000	2.68	0.001	3.27	0.000
	13	1.75	0.055	2.23	0.008	1.53	0.064	1.82	0.007
G161900	2	1.72	0.713	6.07	0.170	1.22	0.862	2.43	0.272
	3	2.07	0.066	2.99	0.068	1.31	0.643	2.01	0.107
	4	3.45	0.218	2.33	0.243	16.47	0.008	5.09	0.047
	5	2.85	0.019	3.41	0.013	5.39	0.025	3.57	0.001
	7	14.23	0.120	1.35	0.836	5.07	0.314	4.60	0.149
	9	0.11	0.126	0.12	0.234	0.36	0.253	0.18	0.074

	11	0.37	0.518	0.91	0.954	0.09	n.a	0.44	0.486
	13	7.87	n.a	2.38	n.a	9.73	n.a	4.86	n.a
G163901	2	0.92	0.524	1.40	0.167	1.08	0.493	1.14	0.403
	3	1.30	0.078	1.15	0.510	0.70	0.108	1.01	0.946
	4	1.66	0.118	1.93	0.001	2.27	0.003	1.94	0.003
	5	1.68	0.003	1.52	0.064	0.97	0.866	1.41	0.079
	7	3.87	0.082	2.42	0.053	1.16	0.711	2.22	0.096
	9	1.48	0.103	1.91	0.015	1.72	0.160	1.69	0.031
	11	2.33	0.048	1.76	0.282	1.44	0.521	1.81	0.129
	13	2.94	0.025	4.27	0.010	3.58	0.017	3.56	0.001
G164800	2	1.26	0.387	0.99	0.948	0.83	0.371	0.78	0.908
	3	0.97	0.922	1.52	0.052	1.34	0.198	1.65	0.227
	4	1.12	0.322	1.00	0.972	1.01	0.895	1.05	0.615
	5	0.93	0.717	0.74	0.143	0.81	0.359	0.75	0.174
	7	1.15	0.550	1.04	0.870	0.87	0.421	1.13	0.943
	9	0.40	0.008	0.58	0.014	0.77	0.339	0.48	0.023
	11	0.78	0.172	0.59	0.059	0.81	0.281	0.99	0.048
	13	0.82	0.360	0.40	0.003	0.77	0.384	0.51	0.077
G165600	3	1.02	0.872	1.22	0.099	0.88	0.306	1.03	0.797
	5	0.76	0.072	0.57	0.016	0.97	0.859	0.73	0.055
	7	0.58	0.054	0.70	0.065	0.95	0.746	0.73	0.076
	9	0.80	0.254	0.75	0.172	1.05	0.830	0.85	0.279
	11	1.14	0.439	1.10	0.603	1.15	0.521	1.13	0.387
	13	0.91	0.657	0.82	0.440	0.99	0.954	0.91	0.513
G165900	3	1.38	0.148	1.06	0.784	1.46	0.113	1.29	0.082
	5	0.88	0.576	0.60	0.011	1.10	0.496	0.81	0.278
	7	0.34	0.006	0.41	0.003	1.55	0.121	0.59	0.229
	9	0.25	0.000	0.26	0.000	0.40	0.096	0.30	0.001
	11	0.43	0.003	0.24	0.001	0.38	0.005	0.34	0.000
	13	n.a.	n.a.	0.37	0.050	0.45	0.107	0.40	0.016
G166300	3	1.13	0.420	1.26	0.064	1.15	0.212	1.18	0.137
	5	1.33	0.000	0.84	0.074	1.11	0.124	1.07	0.581
	7	1.56	0.050	1.14	0.067	1.08	0.309	1.24	0.117
	9	0.98	0.879	1.00	1.000	0.90	0.497	0.96	0.692
	11	1.17	0.342	1.30	0.133	1.03	0.870	1.16	0.194
	13	1.51	0.023	1.28	0.186	1.21	0.118	1.33	0.043

DAP, days after pod setting
n.a. data not available

Table S5. Orthologous genes putatively involved in pathways associated to pod shattering modulation across different species.

Orthologous Genes	Phenotype/evidences of convergent evolution
PvMYB26 (*P. vulgaris*), ***VaMYB26*** (*V. angularis*), ***VuMYB26*** (*V. unguiculata*)	QTL mapping for pod shattering and parallel analysis of gene expression and pod anatomy (*P. vulgaris*; this research) and GWAS for pod shattering (*P. vulgaris*; Rau *et al.*, 2019, Parker *et al.*, 2020); QTL mapping and association with pod shattering (*V. angularis*, Takahashi *et al.*, 2019b) and pod tenderness modulation (*V. unguiculata,* Takahashi *et al.*, 2019b) and QTL mapping for pod shattering (*V. unguiculata*; Lo et *al.*, 2018).
GmPDH1 (*G. max*), ***PDH1*** (*P. vulgaris*)	Correlation with lignin pattern deposition in the sclerenchyma of pod valves and pod shattering modulation by promoting torsion of dried pod walls (*G. max*; Funatsuki *et al.*, 2014); the presence of a putative causal polymorphism in the gene is associated with low pod shattering susceptibility (*P. vulgaris*; Parker *et al.*, 2020), selection signatures (*P. vulgaris*; Schmutz *et al.*, 2014) and expression data (*P. vulgaris*; this research)
PvIND (*P.vulgaris*), ***AtIND*** (*A. thaliana*)	Valve margin lignification and silique shattering modulation (*A. thaliana*; Liljegren *et al.*, 2004); co-mapping with the *St* locus for the presence of pod string (*P. vulgaris*; Koinange *et al.*, 1996) and expression data (*P. vulgaris*; this research)
**Phvul.010G118700** (*P. vulgaris*), ***NST1*** (*A. thaliana*), ***GmSHAT1-5*** (*G. max*)	Formation of secondary cell walls at the valve margin that is required for silique shattering (*A. thaliana*; Mitsuda and Ohme-Takagi 2008); cell wall thickening in the fiber cap cells and pod shattering resistance (*G. max*; Dong *et al.*, 2014); putative candidate for shattering-related functions based on the expression pattern (*P. vulgaris*; this research) and selection signatures (*P. vulgaris*; Bellucci *et al.*, 2014)
**Phvul.009G203400** (*P. vulgaris*), ***AtFUL*** (*A. thaliana*)	Regulation of the valve identity and co-regulation with genes involved in silique shattering (*A. thaliana*; Gu *et al.*, 1998, see also Dong and Wang 2015 for review); QTL mapping for pod shattering modulation (*P. vulgaris*; Rau *et al.*, 2019 and Parker *et al.*, 2020) and signatures of parallel domestication in Andean and Mesoamerican pools (*P. vulgaris*; Schmutz *et al.*, 2014 and Bellucci *et al.*, 2014),

Table S6. Sequences of the single-stranded oligos for the adapters used for GBS library preparation. _F, _R, forward and reverse oligo sequences of the 24 barcoded-adapters are provided. In bold, barcodes are highlighted.

Ss Adapter ID	Ss Adapter Sequence 5'-3'
Tag1_F	ACACTCTTTCCCTACACGACGCTCTTCCGATCT**AACAA**
Tag1_R	/5Phos/CWG**TTGTT**AGATCGGAAGAGCACACGTCT
Tag2_F	ACACTCTTTCCCTACACGACGCTCTTCCGATCT**CCACC**
Tag2_R	/5Phos/CWG**GGTGG**AGATCGGAAGAGCACACGTCT
Tag3_F	ACACTCTTTCCCTACACGACGCTCTTCCGATCT**TTGTT**
Tag3_R	/5Phos/CWG**AACAA**AGATCGGAAGAGCACACGTCT
Tag4_F	ACACTCTTTCCCTACACGACGCTCTTCCGATCT**GGTGA**
Tag4_R	/5Phos/CWG**TCACC**AGATCGGAAGAGCACACGTCT
Tag5_F	ACACTCTTTCCCTACACGACGCTCTTCCGATCT**AACAGT**
Tag5_R	/5Phos/CWG**ACTGTT**AGATCGGAAGAGCACACGTCT
Tag6_F	ACACTCTTTCCCTACACGACGCTCTTCCGATCT**CCATGA**
Tag6_R	/5Phos/CWG**TCATGG**AGATCGGAAGAGCACACGTCT
Tag7_F	ACACTCTTTCCCTACACGACGCTCTTCCGATCT**TTGCCA**
Tag7_R	/5Phos/CWG**TGGCAA**AGATCGGAAGAGCACACGTCT
Tag8_F	ACACTCTTTCCCTACACGACGCTCTTCCGATCT**ACTGTT**
Tag8_R	/5Phos/CWG**AACAGT**AGATCGGAAGAGCACACGTCT
Tag9_F	ACACTCTTTCCCTACACGACGCTCTTCCGATCT**GGAACGT**
Tag9_R	/5Phos/CWG**ACGTTCC**AGATCGGAAGAGCACACGTCT
Tag10_F	ACACTCTTTCCCTACACGACGCTCTTCCGATCT**CACCTGA**
Tag10_R	/5Phos/CWG**TCAGGTG**AGATCGGAAGAGCACACGTCT
Tag11_F	ACACTCTTTCCCTACACGACGCTCTTCCGATCT**CTTGAAT**
Tag11_R	/5Phos/CWG**ATTCAAG**AGATCGGAAGAGCACACGTCT
Tag12_F	ACACTCTTTCCCTACACGACGCTCTTCCGATCT**TCGTGTA**
Tag12_R	/5Phos/CWG**TACACGA**AGATCGGAAGAGCACACGTCT
Tag13_F	ACACTCTTTCCCTACACGACGCTCTTCCGATCT**GGAACGGT**
Tag13_R	/5Phos/CWG**ACCGTTCC**AGATCGGAAGAGCACACGTCT
Tag14_F	ACACTCTTTCCCTACACGACGCTCTTCCGATCT**AACCTAGA**
Tag14_R	/5Phos/CWG**TCTAGGTT**AGATCGGAAGAGCACACGTCT
Tag15_F	ACACTCTTTCCCTACACGACGCTCTTCCGATCT**CTTGATGA**
Tag15_R	/5Phos/CWG**TCATCAAG**AGATCGGAAGAGCACACGTCT
Tag16_F	ACACTCTTTCCCTACACGACGCTCTTCCGATCT**AGGTCGGT**
Tag16_R	/5Phos/CWG**ACCGACCT**AGATCGGAAGAGCACACGTCT
Tag17_F	ACACTCTTTCCCTACACGACGCTCTTCCGATCT**TAACGAACA**
Tag17_R	/5Phos/CWG**TGTTCGTTA**AGATCGGAAGAGCACACGTCT
Tag18_F	ACACTCTTTCCCTACACGACGCTCTTCCGATCT**GCCAACCAT**
Tag18_R	/5Phos/CWG**ATGGTTGGC**AGATCGGAAGAGCACACGTCT
Tag19_F	ACACTCTTTCCCTACACGACGCTCTTCCGATCT**CTTGTGTTA**
Tag19_R	/5Phos/CWG**TAACACAAG**AGATCGGAAGAGCACACGTCT

Tag20_F	ACACTCTTTCCCTACACGACGCTCTTCCGATCT**ACGTGTGGT**
Tag20_R	/5Phos/CWG**ACCACACGT**AGATCGGAAGAGCACACGTCT
Tag21_F	ACACTCTTTCCCTACACGACGCTCTTCCGATCT**TGAACACAA**
Tag21_R	/5Phos/CWG**TTGTGTTCA**AGATCGGAAGAGCACACGTCT
Tag22_F	ACACTCTTTCCCTACACGACGCTCTTCCGATCT**GACCACACT**
Tag22_R	/5Phos/CWG**AGTGTGGTC**AGATCGGAAGAGCACACGTCT
Tag23_F	ACACTCTTTCCCTACACGACGCTCTTCCGATCT**CTTGGTTGA**
Tag23_R	/5Phos/CWG**TCAACCAAG**AGATCGGAAGAGCACACGTCT
Tag24_F	ACACTCTTTCCCTACACGACGCTCTTCCGATCT**ACGTTGGTT**
Tag24_R	/5Phos/CWG**AACCAACGT**AGATCGGAAGAGCACACGTCT

Table S7. Sequences of the primers used for the amplification, indexing and quantification of the GBS library. In bold, Illumina indexes are highlighted.

**Primer ID**	**Primer Sequences 5'-3'**
PPI1 Illumina Index	CAAGCAGAAGACGGCATACGAGAT**CGTGAT**GTGACTGGAGTTC
PPI2 Illumina Index	CAAGCAGAAGACGGCATACGAGAT**ACATCG**GTGACTGGAGTTC
PPI3 Illumina Index	CAAGCAGAAGACGGCATACGAGAT**GCCTAA**GTGACTGGAGTTC
PPI8 Illumina Index	CAAGCAGAAGACGGCATACGAGAT**TCAAGT**GTGACTGGAGTTC
PPI9 Illumina Index	CAAGCAGAAGACGGCATACGAGAT**CTGATC**GTGACTGGAGTTC
PPI10 Illumina Index	CAAGCAGAAGACGGCATACGAGAT**AAGCTA**GTGACTGGAGTTC
PPI11 Illumina Index	CAAGCAGAAGACGGCATACGAGAT**GTAGCC**GTGACTGGAGTTC
PPI25 Illumina Index	CAAGCAGAAGACGGCATACGAGAT**ATCAGT**GTGACTGGAGTTC
MP1 Primer	AATGATACGGCGACCACCGAGATCTACACTCTTTCCCTACACGACGCTCTTCCGATCT
MP2 Primer	GTGACTGGAGTTCAGACGTGTGCTCTTCCGATCT
qPCR Primer 1.1	ATTGATACGGCGACCACCGAGAT
qPCR Primer 2.1	CAAGCAGAAGACGGCATACGA

Supplementary dataset

Dataset S1. List of accessions that were grown for pod collection, RNA-seq and differential gene expression analyses.

Dataset S2. Significant SNPs identified across different GWAS mapping experiment at the major locus *qPD5.1-Pv* for loss of pod shattering. Name, location, and level of significance are reported for each SNP. The position of each SNP (i.e., genic or intergenic) and information on the physically closest genes are reported. NA, Gene description not available

Dataset S3. Genes identified within the major locus *qPD5.1-Pv* for loss of pod shattering. Details of gene name and location, and description of common bean genes are reported. Genes with selection signature in Schmutz *et al.* (2014) and Bellucci *et al.* (2014) are highlighted. The $\log_2$ fold-change (differential expression for RNA-seq data) is shown for each comparison. Significance: red shading, $p < 0.001$. DE, differential expression (in at least one comparison); PS, putatively under selection. N.A., gene description not available; NA, differential expression cannot be calculated (no gene expression was detected for the compared samples); n.m., no reads (for RNA-seq data) and no contigs (for identification of genes putatively under selection) mapped for the gene.

Dataset S4. Genes in common bean that are orthologous to genes in other species with known functions that are putatively involved in seed shattering or have potentially related functions (e.g., cell-wall modification, differentiation). Details of gene name and location, and description of common bean genes are reported. Genes with selection signature in Schmutz *et al.* (2014) and Bellucci *et al.* (2014) are indicated. The $\log_2$ fold-changes (differential expression for RNA-seq data) is shown for each comparison. Significance: red shading, $p < 0.001$. References and sources for the identification of the orthologues are reported. N.A., gene description not available; NA, differential expression cannot be calculated (no gene expression was detected for the compared samples); n.m., no reads (for RNA-seq data) and no contigs (for identification of genes putatively under selection) mapped for the gene.

Dataset S5. Genes in common bean that are putatively involved in the phenylpropanoid biosynthesis pathway. Genes were identified based on gene descriptions available on Phytozome and the Plant Metabolic Network database, and according to the function of the orthologous genes in soybean and *A. thaliana*. Genes with selection signature according to Schmutz *et al.* (2014) and Bellucci *et al.* (2014) are indicated. The $\log_2$ fold-changes (differential expression for RNA-seq data) is shown for each comparison. Significance: red shading, $p < 0.001$. NA, enzymatic reaction information not available, or differential expression cannot be calculated (no gene expression was detected for the compared samples); n.m., no reads (for RNA-seq data) and no contigs (for identification of genes putatively under selection) mapped for the gene.

Supplementary Protocol S1.

Protocol for the GBS library preparation

For each sample, 200 ng of gDNA were digested for 2 h at 75 °C with 1.25 U ApeKI (New England Biolabs, NEB) in 1X NEB 3.1 buffer, in a final volume of 20 µL. The results of the digestion were verified by running the digested DNA and the intact gDNA on a 4200 TapeStation using the Genomic DNA assay (Agilent Technologies). The digested DNA was ligated to a double-stranded barcoded-adapter (previously annealed, 0.05µM final concentration) with 1 U T4 DNA ligase (Invitrogen) in the presence of 1X ligase buffer in a final volume of 50µl. A total of 24 different barcoded-adapters were employed to uniquely identify 24 samples at a time (Table S6). The ligation reaction was performed in a thermocycler for 10 min at 30 °C, and 4 h at 22 °C (unheated lid), followed by inactivation for 30 min at 65 °C (heated lid). The samples were subsequently pooled (25 µL from each sample; 24 samples with different barcoded-adapters) and purified using beads (0.4X AMPure XP; Beckman Coulter) following the manufacturer's instructions. The purified pool was resuspended in 30µl water. The DNA fragments with the desired length were selected using a BluePippin system (Sage Science). The 30µl purified pool was loaded in a 1.5% Agarose Dye-Free cassette (internal standard, 250bp-1.5kb DNA size range) and run with a tight mode set to 550 bp. The eluted size-selected pool (around 40-50 µl) was brought to a volume of 60µl with water. Half of the purified and size-selected pool (30µl) was subsequently amplified in 50µl reaction volume using 1 U Taq Phusion polymerase in the presence of 1X Taq Phusion HF buffer, 0.3 mM dNTPs and three different primers: Primer MP1 (0.5µM), Primer MP2 (0.01µM) and PPIX Illumina Index (0.5µM), the latter including an index for Illumina sequencing. A total of eight PPI Illumina Index primers with eight different Illumina indexes were utilized, allowing a multiplexing of 8 pools (=192 samples) at a time. Primer sequences are reported in Table S7. Amplification was performed following the PCR programme of: 30 s at 98 °C, 18 cycles of 10 s at 98 °C, 30 s at 65 °C and 30 s at 72 °C, and 5 mins at 72 °C for final elongation. Final GBS libraries were purified with beads (1.5X AMPure XP; Beckman Coulter). The size distribution of final GBS libraries was performed on a 4200 TapeStation using a D1000 Assay (the average size distribution expected was 560bp). Figure S13 shows the final GBS library structure. The final GBS libraries were quantified by qPCR using primers annealing on the Illumina adapter sequences (Table S7), and on the basis of a reference standard curve.

References

Battat, M. *et al.* (2019). A MYB triad controls primary and phenylpropanoid metabolites for pollen coat patterning. *Plant Physiology* 180, 87–108.

Berthet, S. *et al.* (2011) Disruption of LACCASE4 and 17 results in tissue-specific alterations to lignification of *Arabidopsis thaliana* stems. *The Plant Cell* 23, 1124–1137.

Geng, P. *et al.* (2019). MYB20, MYB42, MYB43 and MYB85 regulate phenylalanine and lignin biosynthesis during secondary cell-wall formation. *Plant Physiology* 182, 1272–1283.

Hao, Z. *et al.* (2014). Loss of *Arabidopsis* GAUT12/IRX8 causes anther indehiscence and leads to reduced G lignin associated with altered matrix polysaccharide deposition. *Frontiers in Plant Science* 5, 357.

Johnson, K.L., Kibble, N.A.J., Bacic, A. and Schultz, C.J. (2011). A fasciclin-like arabinogalactan-protein (FLA) mutant of *Arabidopsis thaliana*, *fla1*, shows defects in shoot regeneration. *PLoS ONE* 6(9), e25154.

Kim, W. *et al.* (2013). MYB46 directly regulates the gene expression of secondary wall-associated cellulose synthases in *Arabidopsis*. *The Plant Journal* 73, 26–36.

Ko, J., Kim, W. and Han, K. (2009). Ectopic expression of MYB46 identifies transcriptional regulatory genes involved in secondary wall biosynthesis in Arabidopsis. *The Plant Journal* 60, 649–665.

Li, C., Zhou, A. and Sang, T. (2006). Rice domestication by reducing shattering. *Science* 311, 1936–1939.

Li, W., Tian, Z., and Yu, D. (2015). WRKY13 acts in stem development in *Arabidopsis thaliana*. *Plant Science* 236, 205–213.

Lin, Z. *et al.* (2007). Origin of seed shattering in rice (*Oryza sativa* L.). *Planta* 226, 11–20.

Lin, Z. *et al.* (2012). Parallel domestication of the *Shattering1* genes in cereals. *Nature Genetics* 44, 720–724.

Mao, L. *et al.* (2000). *JOINTLESS* is a MADS-box gene controlling tomato flower abscission zone development. *Nature* 406, 910–913.

McCarthy, R.L., Zhong, R. and Ye, Z-H. (2009). MYB83 Is a direct target of SND1 and acts redundantly with MYB46 in the regulation of secondary cell-wall biosynthesis in *Arabidopsis*. *Plant and Cell Physiology* 50, 1950–1964.

Nakano, T. *et al.* (2012). MACROCALYX and JOINTLESS interact in the transcriptional regulation of tomato fruit abscission zone development. *Plant Physiology* 158, 439–450.

Newman, L.J., Perazza, D.E., Juda, L. and Campbel, M.M. (2004). Involvement of the R2R3-MYB, *At*MYB61, in the ectopic lignification and dark-photomorphogenic components of the *det3* mutant phenotype. *The Plant Journal* 37, 239–250.

Ogawa, M., Kay, P., Wilson, S. and Swain S.M. (2009). ARABIDOPSIS DEHISCENCE ZONE POLYGALACTURONASE1 (ADPG1), ADPG2, and QUARTET2 are polygalacturonases

required for cell separation during reproductive development in *Arabidopsis*. *The Plant Cell* 21, 216–233.

Persson, S. *et al.* (2007). The *Arabidopsis irregular xylem8* mutant is deficient in glucuronoxylan and homogalacturonan, which are essential for secondary cell-wall integrity. *The Plant Cell*, 19, 237–255.

Preston, J., Wheeler, J., Heazlewood, J., Li, S.F. and Parish, R.W. (2004). AtMYB32 is required for normal pollen development in *Arabidopsis thaliana*. *The Plant Journal* 40, 979–995.

Rajani, S. and Sundaresan, V. (2001). The *Arabidopsis* myc-bHLH gene *ALCATRAZ* enables cell separation in fruit dehiscence. *Current Biology* 11, 1914–1922.

Roeder, A.H.K., Ferrándiz, C. and Yanofsky, M.F. (2003). The role of the REPLUMLESS homeodomain protein in patterning the *Arabidopsis* fruit. *Current Biology* 13, 1630–1635.

Romano, J.M. *et al.* (2012). *At*MYB61, an R2R3-MYB transcription factor, functions as a pleiotropic regulator via a small gene network. *New Phytologist* 195, 774–786.

Shin, B. *et al.* (2002). *AtMYB21*, a gene encoding a flower-specific transcription factor, is regulated by COP1. *The Plant Journal* 30, 23–32.

Sorefan, K. *et al.* (2009). A regulated auxin minimum is required for seed dispersal in *Arabidopsis*. *Nature* 459, 583–586.

Taylor, N.G., Gardiner, J.C., Whiteman, R. and Turner, S.R. (2004). Cellulose synthesis in the *Arabidopsis* secondary cell wall. *Cellulose* 11, 329–338.

Wei, P. *et al.* (2010). Overexpression of AtDOF4.7, an *Arabidopsis* DOF family transcription factor, induces floral organ abscission deficiency in *Arabidopsis*. *Plant Physiology* 153, 1031–1045.

Yamaguchi, M. *et al.* (2010). VND-INTERACTING2, a NAC domain transcription factor, negatively regulates xylem vessel formation in *Arabidopsis*. *The Plant Cell* 22, 1249–1263.

Zhao, Q. *et al.* (2013). *LACCASE* is necessary and nonredundant with *PEROXIDASE* for lignin polymerization during vascular development in *Arabidopsis*. *The Plant Cell* 25, 3976–3987.

Zhong, R. and Ye, Z-H. (2012). MYB46 and MYB83 bind to the SMRE sites and directly activate a suite of transcription factors and secondary wall biosynthetic genes. *Plant and Cell Physiology* 53, 368–380.

Zhong, R., Richardson, E.A. and Ye, Z-H. (2007). The MYB46 transcription factor is a direct target of SND1 and regulates secondary wall biosynthesis in *Arabidopsis*. *The Plant Cell* 19, 2776–2792.

Zhou, J., Lee, C., Zhong, R. and Ye, Z-H. (2009). MYB58 and MYB63 are transcriptional activators of the lignin biosynthetic pathway during secondary cell-wall formation in *Arabidopsis*. *The Plant Cell* 21, 248–266.

Zhou, J., Zhong, R. and Ye, Z-H. (2014). Arabidopsis NAC domain proteins, VND1 to VND5, are transcriptional regulators of secondary wall biosynthesis in vessels. *PLoS ONE* 9, e105726.

Zhou, M. *et al*. (2017). LNK1 and LNK2 co-repressors interact with the MYB3 transcription factor in phenylpropanoid biosynthesis. *Plant Physiology* 174, 1348–1358.

Zhou, Y. *et al.* (2012). Genetic control of seed shattering in rice by the APETALA2 transcription factor *SHATTERING ABORTION1*. *The Plant Cell* 24, 1034–1048.
